# Comparative Effectiveness of Di'ao Xin Xue Kang Capsule and Compound Danshen Tablet in Patients With Symptomatic Chronic Stable Angina

**DOI:** 10.1038/srep07058

**Published:** 2014-11-14

**Authors:** Yanan Yu, Siyuan Hu, Guoxin Li, Jie Xue, Zhuoming Li, Xiangling Liu, Xiyan Yang, Bo Dong, Donghai Wang, Xiaofeng Wang, Shurong Liu, Jun Liu, Bingwei Chen, Liying Wang, Songshan Liu, Qiguang Chen, Chunti Shen, Zhong Wang, Yongyan Wang

**Affiliations:** 1 Institute of Basic Research in Clinical Medicine, China Academy of Chinese Medical Sciences, No. 16 Nanxiaojie, Dongzhimen nei, Beijing, 100700, China; 2 First Teaching Hospital of Tianjin University of Traditional Chinese Medicine, AnShan Xi Road 314, Nankai District, 300193, Tianjin, China; 3 The Second Hospital Affiliated to Liaoning University of TCM, HuangHe North Road 60, Huanggu District, Shenyang 110034, Liaoning, China; 4 The TCM Hospital of Xinjiang Uygur Autonomous Region, HuangHe Road 116, Wulumuqi 830099, Xinjiang, China; 5 The Jilin Provincial Hospital of Integrated TCM and Western Medicine, Gongnong Da Road 1745, Chaoyang District, Changchun 130021, Jilin, China; 6 School of Public Health, Southeast University, Dijia Qiao 87, Nanjing 210009, Jiangsu, China; 7 The Affiliated Hospital of Chengdu University of Traditional Chinese Medicine, No.37 Twelve Road, Chengdu 610075, Sichuan, China; 8 Changzhou TCM Hospital, Heping North Road, Tianing District, Changzhou 213004, Jiangsu, China

## Abstract

A high proportion of patients with stable angina remains symptomatic despite multiple treatment options. Di'ao Xinxuekang (XXK) capsule and Compound Danshen (CDS) tablet have been approved for treating angina pectoris for more than 20 years in China. We compare the anti-anginal effectiveness of XXK capsule and CDS tablet in patients with symptomatic chronic stable angina. A randomized, multicenter, double-blind, parallel-group, superiority trial was conducted in 4 study sites. 733 patients with symptomatic chronic stable angina were included in the full analysis set. The primary outcomes were the proportion of patients who were angina-free and the proportion of patients with normal electrocardiogram (ECG) recordings during 20 weeks treatment. Compared with CDS, XXK significantly increased the proportion of angina-free patients, but no significant difference was noted in the proportion of patients with normal ECG recordings. Weekly angina frequency and nitroglycerin use were significantly reduced with XXK versus CDS at week 20. Moreover, XXK also improved the quality of life of angina patients as measured by the SAQ score and *Xueyu Zheng* (a type of TCM syndrome) score. We demonstrate that XXK capsule is more effective for attenuating anginal symptoms and improving quality of life in patients with symptomatic chronic stable angina, compared with CDS tablet.

Nearly 58% of patients with coronary artery disease were suffering from chronic stable angina^1^. Current treatment strategies aim to reduce the risk of mortality and morbid events and to reduce symptoms^2^. For patients, it is often the latter that is of greater concern^3^. Despite multiple treatment options including pharmacotherapy (as organic nitrates, β-blockers, calcium channel antagonists), revascularization, lifestyle management and several alternative procedures^2^,^3^,^4^, a high proportion of patients with stable angina remains symptomatic and their quality of life is impaired^5^,^6^,^7^. Moreover, several observational studies have shown that angina symptoms such as physical limitation and angina frequency are predictive of mortality and acute coronary syndrome (ACS) hospitalizations^8^,^9^,^10^. Thus, the potential role of patient-centered symptomatic medical treatment warrants further consideration^5^. Translational medicine guides modern medical research toward a patient-centered outcomes research and evidence-based clinical decisions^11^. Bioactive components derived from herbal medicines including Traditional Chinese Medicine (TCM) seems to be promising in this respect. Through long clinical practice in the real world, TCM based on a sophisticated system of medical theory and focused on disease status and response to treatment^12^, as well as the quality-of-life of patients^13^. The efficacy of some herbal medicine has been documented in several well-designed randomized studies^14^,^15^, and the treatment of angina is also frequently used in the practice of TCM^16^,^17^.

As a post-marketing herbal product in China and Netherlands, the active ingredient (Dioscin) of Di'ao Xinxuekang capsule (XXK) is extracted from the rhizomes of *Dioscorea panthaica Prain et Burkill*^18^, which has been demonstrated to prevent and treat coronary diseases by increasing blood flow and enhancing oxygen delivery to the ischaemic myocardium via vasodilatation of the coronary vasculature^19^; reducing myocardial oxygen consumption by decreasing preload and afterload^20^; maintaining the activity of Ca^2+^ -ATP enzyme and Na^+^- K^+^ -ATP enzyme through free radicals removal^21^; and protecting cardiac cells from ischemia/reperfusion injury by preventing apoptosis and modulating the mitochondrial apoptotic pathway through attenuation of oxidative stress^22^. Besides, previous clinical trials conducted in China have shown that XXK can reduce anginal symptoms and improve myocardial ischemia, compared with nitroglycerin or other antianginal drugs^23^,^24^,^25^. But challenges and uncertainties still exist: the methodological quality of previous studies is generally low^26^,^27^, and the long-term effectiveness and safety of XXK for the management of chronic stable angina have not been validated.

Compound Danshen (CDS) tablet, another post-marketing herbal product approved by China Food and Drug Administration (CFDA), consisting of *Savia miltiorrhiza, Panax Notoginseng* and *Borneol*, is officially recorded in Chinese Pharmacopoeia^28^ and also widely used to treat ischemic heart diseases in China. Multiple main active ingredient of CDS tablet has been demonstrated to possess therapeutic effects in the cardiovascular diseases^29^,^30^,^31^. CDS tablet has also been proven to be effective in dilating coronary arteries and decreasing myocardial oxygen consumption^32^.

In this study, we conducted a randomized superiority trial to investigate the health benefits of XXK capsule compared with CDS tablet, and hypothesized that XXK would induce a greater increase in the proportion of angina-free patients at the end of the 20-week intervention period.

## Results

### Baseline characteristics

**Figure 1:**
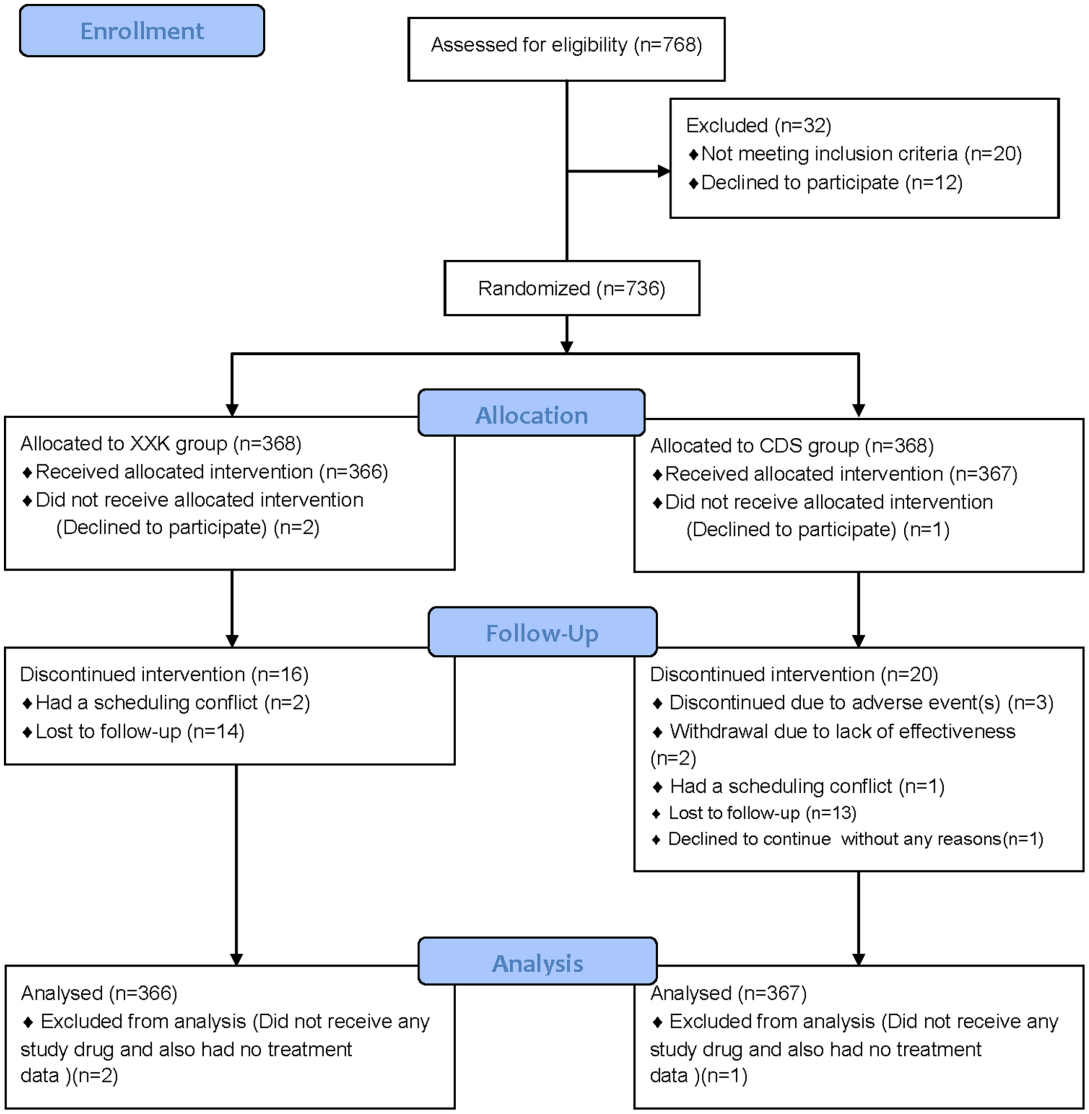
The flow diagram of the trial. XXK = Xinxuekang; CDS = Compound Danshen.

From February 14th, 2009 through September 21st, 2011, 768 patients were screened (Figure 1). Of these, 736 patients were considered trial-eligible and then randomly assigned in equal numbers to the XXK group or the CDS group. Two XXK patients and 1 CDS patient were excluded from the full analysis set because they did not receive any study drug and also had no treatment data. Finally, a total of 733 patients were included in the full analysis set, 366 in the XXK group and 367 in the CDS group. The rates on the discontinued intervention were similar between the two groups (4.37% in XXK group vs. 5.45% in CDS group, *P* = 0.50). The baseline characteristics of the study groups are shown in Table 1. The distributions of the demographic and clinical characteristics between the two groups were reasonably well-balanced, except that the mean heart rate in the XXK group was lower than that in the CDS group (70.20 ± 8.65 vs. 71.62 ± 8.77, *P* = 0.0285). There were no significant differences in eligible patients with concomitant diseases including diabetes mellitus [46(12.57%) vs. 55(14.99%), *P* = 0.3423], hypertension [19(5.19%) vs. 28(7.63), *P* = 0.1779] or hyperlipidemia [25(6.83%) vs. 26(7.08%), *P* = 0.8926] as well as the use of concomitant medications including aspirin or clopidogrel [5(1.37%) vs. 7(1.91%), *P* = 0.5637], antihypertensive [39(10.66%) vs. 48(13.08%), *P* = 0.3105], antihyperlipidemic [2(0.55%) vs. 7(1.91%), *P* = 0.1811] or antidiabetics [20(5.46%) vs. 27(7.36%), *P* = 0.2957] drugs between XXK and CDS groups.

### Primary outcomes

**Figure 2:**
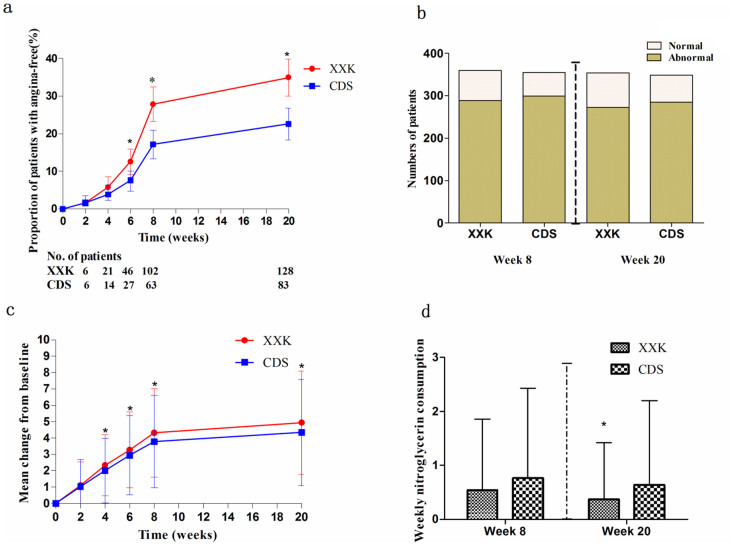
Changes in primary outcomes, weekly angina frequency, and nitroglycerin use. (a) Angina-free patients: P = 0.0185 at week 6, 0.0005 at week 8, 0.0002 at week 20.* p < 0.025. (b) Patients with normal ECG recordings: P = 0.1919 at week 8, 0.0907 at week 20. (c) Weekly angina frequency. *p < 0.05. (d) Weekly nitroglycerin use. *p < 0.05. ECG = electrocardiogram.

**Figure 3:**
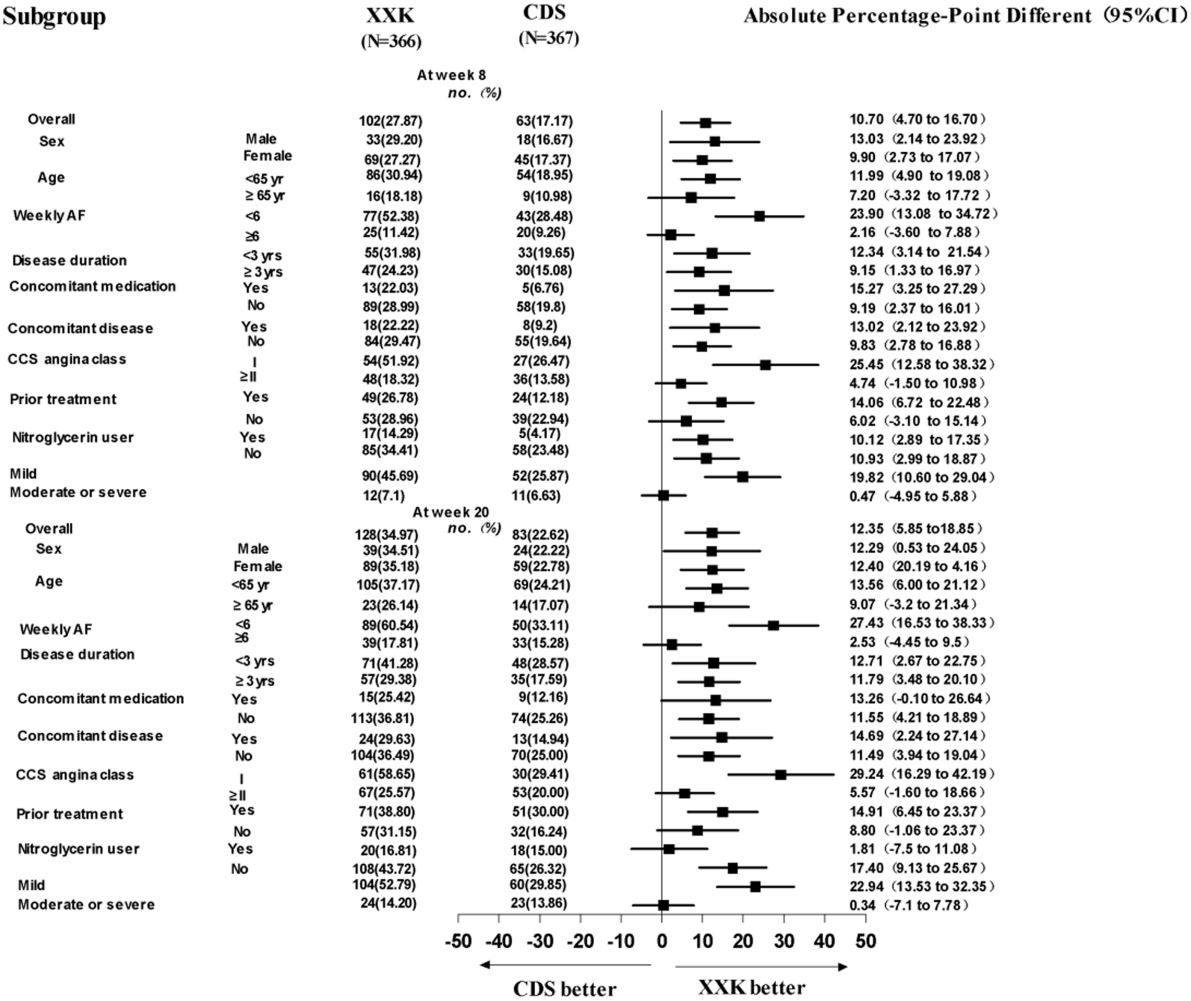
Subgroup analyses for the primary endpoint (angina-free) at week 8 and week 20. CCS = Canadian Cardiovascular Society; AF = Angina frequency.

In both groups, the number and proportion of patients who became angina-free gradually increased during the intervention period. It is shown in Figure 2a that from week 6 until week 20, the XXK group had a significantly greater increase in the proportion of angina-free patients as compared with the CDS group (12.57% [95%CI, 9.17 to 15.96] vs. 7.36% [95%CI, 4.69 to 10.03] at week 6, P = 0.0185; 27.87% [95% CI, 23.28 to 32.46] vs. 17.17% [95%CI, 13.31 to 21.02] at week 8, *P* = 0.0005; 34.97% [95%CI, 30.09 to 39.86] vs. 22.62% [95%CI, 18.34 to 26.90] at week 20, *P* = 0.0002). Results from the effectiveness analyses by subgroups for the primary endpoint (angina-free) at week 8 and week 20 are shown in Figure 3. Findings from these subgroup analyses were generally consistent with those obtained from the entire study population, except in patients aged ≥65 years, with a weekly angina frequency ≥6 episodes, Canadian Cardiovascular Society (CCS) angina class ≥ II, without prior treatment, and with moderate or severe angina symptoms, where no significant differences were identified between the two groups.

The proportion of patients with normal electrocardiogram (ECG) recordings tended to increase both in the XXK group (from 0.55% at baseline to 19.50% at week 8, and then 22.84% at week 20) and the CDS group (from 0.55% to 15.77%, and then 17.75%), but no significant differences were observed between groups (Figure 2b).

### Secondary outcomes

Though patients treated with CDS had a substantial change from baseline in weekly angina frequency, the change was significantly greater in the XXK group, beginning from week 4 until week 20 (Figure 2c; *P* = 0.0213 − 0.0127). At week 20, the XXK group had a significantly lower weekly angina frequency compared with the CDS group (mean 2.01 ± 1.95 vs. 2.58 ± 2.07, respectively; *P* = 0.0001).

The average weekly nitroglycerin consumption rate in the XXK group was similar with that of the CDS group at week 8 (0.54 ± 1.32 vs. 0.77 ± 1.66, respectively; *P* = 0.1111), but then reached statistical significance at week 20 (0.37 ± 1.05 vs. 0.64 ± 1.56, respectively; *P* = 0.0322) (Figure 2d).

**Figure 4:**
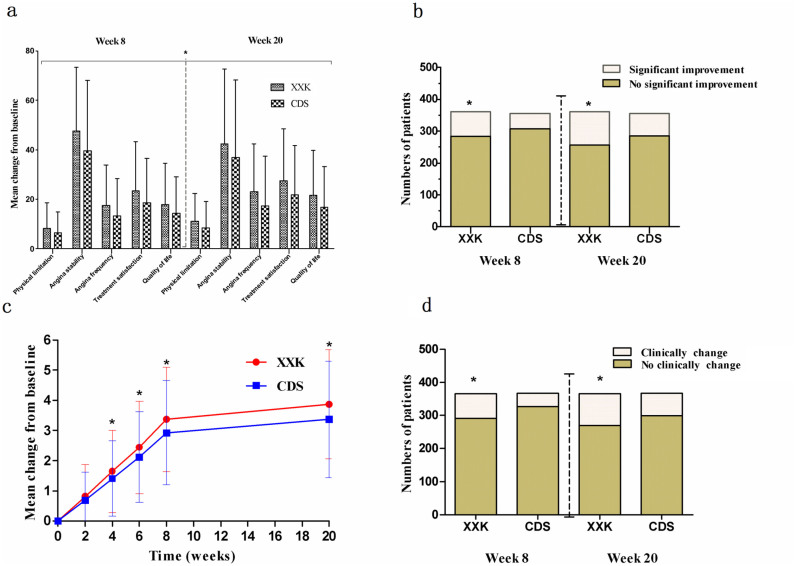
Comparisons of SAQ questionnaire and *Xueyu Zheng* scores between XXK and CDS groups. (a) Mean change from baseline in 5 domains of SAQ at week 8 and week 20. *p < 0.05. (b) Clinically significant improvements based on SAQ score at week 8 and week 20. *p < 0.05. (c) Change from baseline in *Xueyu Zheng* score. *p < 0.05. (d) Significant syndrome improvements based on *Xueyu Zheng* score at week 8 and week 20. *p < 0.05. SAQ = Seattle Angina Questionnaire.

The changes from baseline in all the 5 dimensions of Seattle Angina Questionnaire (SAQ) scores were significantly improved in the XXK group compared with the CDS group both at week 8 and week 20 (Figure 4a). Meanwhile, the XXK group also had a greater proportion of patients with clinically significant improvements than the CDS group (21.33% [95%CI, 17.10 to 25.56] vs. 13.76% [95%CI, 10.19 to 17.34] at week 8, *P* = 0.0078; 28.81% [95%CI, 24.38 to 33.68] vs. 19.94% [95%CI, 16.12 to 24.91] at week 20, *P* = 0.0057) (Figure 4b). The proportion of patients with significant syndrome improvements based on the *Xueyu Zheng* (a type of TCM syndrome) score was significantly higher in the XXK group than that in the CDS group at week 8 and week 20 (20.22% [95%CI, 16.10 to 24.33] vs. 10.90% [95%CI, 7.71 to 14.09] at week 8, *P* = 0.0005; 26.23% [95%CI, 27.12 to 30.74] vs. 18.53% [95%CI, 14.55 to 22.50] at week 20, *P* = 0.0124) (Figure 4d). We also found that from week 4 to week 20, the differences in the changes from baseline in the *Xueyu Zheng* score reached statistical significance between the two groups (Figure 4c), indicating a superiority of XXK over CDS (*P* = 0.0152 − 0.0003).

At baseline and week 8, Exercise Tolerance Testing (ETT) was administrated in 111 and 110 patients in the XXK and CDS groups, respectively. But no differences were noted between the two groups in the time to onset of ST segment depression, time to onset of angina, and maximum ST segment depression (*P* = 0.5068–0.9273). Also, no differences were identified between the two groups in blood lipid at week 8.

### Safety

Among the 733 patients who received study medications, 19 reported adverse events (AEs). The AEs occurred in 3.54% (13) of CDS− and 1.64% (6) of XXK-treated patients, and most were mild to moderate in severity. The frequencies of these events did not differ significantly between the two groups (*P* = 0.1050). Seven cases of AEs were considered as being possibly related to the study drugs (1 [0.27%] in the XXK group vs. 6 [1.63%] in the CDS group, with no significant difference). This included 1 case of stomach flatulence in the XXK group and 6 cases in the CDS group, including heart burn, abnormal liver function, positive urinary albumin, and oral ulcer. However, all of these changes returned to baseline levels. No serious adverse events occurred in the trial.

## Discussion

In our study, it showed that XXK capsule provided potentially long-term (20 weeks) antianginal benefit in patients with symptomatic chronic stable angina, compared with CDS tablet. The effect was evident in the greater increase in angina-free patients, greater reductions in weekly angina frequency and average weekly nitroglycerin consumption rate, as well as greater improvements in health-related quality of life as compared with the control group. In addition, the rates of AEs were similar in both treatment groups. Most AEs were mild and not considered related to study medication.

Patients without anginal symptoms may have a better prognosis than those with symptoms^33^, and guidelines^3^ on management of chronic stable angina recommend that for most patients, the treatment goal should be complete, or nearly complete, elimination of anginal chest pain. Thus, the performance measure for symptom relief and frequency, as assessed by clinicians or patients, could be an important, patient-centered outcome^34^. The outcome measures used in our study were all patient-centered and clinically meaningful, focusing on absence of anginal symptoms (angina-free), weekly angina frequency, weekly nitroglycerin use, changes in SAQ score and *Xueyu Zheng* score, and the effectiveness of XXK has been well demonstrated. In a recent observational study, patients who experienced weekly angina ≥ 1 were proven to have poorer function and quality of life^33^. In our study, the consistent findings of an increased proportion of angina-free patients at each visit in the XXK group may indicate the potential value of XXK in symptomatic treatment of stable angina. As a critical prognostic indicator^35^, the weekly angina frequency in the XXK group was significantly reduced compared with baseline (reduced by 1.1–4.94 episodes per week from week 2 to week 20), which is similar or even higher than that observed in other antianginal trials using conventional agents^36^,^37^. For example, in the IMAGE study^36^, the standardized therapy with metoprolol and nifedipine reduced angina frequency from a mean of 5 to 7 attacks to an average of 3 to 4 attacks at the end of the study (week 10). In INITIATIVE study (4 months)^37^, the number of angina attacks was decreased by 2.3–2.7 attacks with both ivabradine and atenolol, and the reduction in nitroglycerin use (1.4–1.2 uses per week) was also comparable to the reduction of 1.44 uses per week observed in our study.

Another important finding in this study was the effectiveness of XXK capsule in improving quality of life as assessed by SAQ questionnaire. Our results showed an incremental benefit in the XXK group from week 8 to week 20 in all of the 5 domains as well as the likelihood of a clinically significant improvement from baseline on the basis of the SAQ analyses, while other studies only demonstrated benefits in one or two domains, even when compared with placebo^38^,^39^. Additionally, the superiority of XXK over CDS was also manifested as a greater improvement in the *Xueyu Zheng*, which is considered as a patient-centered outcome particularly in the view of TCM.

As a head-to-head comparative effectiveness research (CER), XXK capsule showed a superior effect than CDS tablet, the results perhaps due to their different active ingredients and pharmacological mechanism. Dioscin, as the active ingredient of XXK capsule protects cardiac cells from ischemia/reperfusion injury by preventing the mitochondrial apoptotic pathway through modulation of the mPTP opening and the regulation of the Bcl-2 family proteins Bax and Bcl-2^40^. While CDS Tablet protect cardiomyocytes against myocardial ischemia and inhibits apoptosis via the Akt-eNOS signaling pathway^41^. Moreover, it may have extensive effects on four metabolites (hypoxanthine, xanthine, inosine and allantoin) in the pathway of purine metabolism which contribute to a decrease of oxygen-free radical^42^.

However, there are several limitations in our study. Firstly, although the association between angina symptoms and mortality has been investigated previously with use of the SAQ^9^,^10^, other measures used in our study such as free of angina, weekly use of nitroglycerin and *Xueyu Zheng* score have not been directly associated with mortality yet. Secondly, the majority of participants were women both in XXK group (69.13%) and CDS group (70.57%), which also potentially limits the generalizability of our results. The unbalanced sex ratio may attribute to both the gender and regional differences that women are more likely to present with angina and have lower pain thresholds during daily life^43^,^44^ and women in northern region have higher incidence of cardiovascular disease in China^45^. Therefore, studies involving a wider population will be conducted to confirm whether XXK capsule could reduce the risk of mortality or fatal cardiac events.

Together, Our trial demonstrates that compared with CDS tablet, Di'ao XXK capsule is more effective for attenuating anginal symptoms and improving quality of life in patients with symptomatic chronic stable angina with a low incidence of AEs. Such findings indicate that XXK capsule may be a more favorable therapeutic option for the long-term management of symptomatic chronic stable angina.

## Methods

### Study design

This was a randomized, multicenter, double-blind, parallel-group, superiority clinical trial, in which subjects with stable angina were randomized to receive either XXK capsule or CDS tablet for 20 weeks.

The study protocol was approved by the Institutional Review Board of the Affiliated Hospital of Nanjing University of Traditional Chinese Medicine (2009NL-004-01). The trial was conducted in 4 study sites across China and the methods were carried out in accordance with the approved guidelines(full protocol and list of participating sites can be found in Supplementary Appendix 1). All patients provided written informed consent before enrollment. An independent Data and Safety Monitoring Board oversaw the conduct of the study and reviewed the safety and effectiveness data. The report of the study adheres to the Consolidated Standards of Reporting Trials (CONSORT) statement and the trial was registered at Chinese Clinical Trial Register (ChiCTR) with the identifier number ChiCTR-TRC-09000332(URL:http://apps.who.int/trialsearch/Trial2.aspx?TrialID=ChiCTR-TRC-09000332).

### Participants

Enrollment occurred from February 2009 through September 2011. Patients aged 40 to 70 years with a confirmed diagnosis of chronic stable angina^46^ were included. Those who suffered from stable effort angina due to coronary heart disease (with angiographic evidence of 60% stenosis of at least 1 major coronary artery, or a stress-induced reversible perfusion defect identified by radionuclide or echocardiographic imaging), CCS angina class I, II, or III^47^ with an attack frequency ≥ two episodes per week and a documented history of ischemic changes on ECG (ST segment depression ≥ 0.05 mv and/or deep T wave inversions > 0.2 mv, or flat T wave less than 1/10R) or positive exercise stress test (defined as occurrence of limiting angina and 1 mm horizontal or downsloping ST-segment depression between 3 and 12 min of initiation) were eligible for inclusion in the study.

Patients were excluded if they had any of the following: severe chronic heart failure, uncontrolled hypertension (systolic BP ≥ 160 mmHg, and/or diastolic BP ≥ 100 mmHg), or cardiac arrhythmias; a history of myocardial infarction, percutaneous coronary intervention, coronary artery bypass grafting or stroke with in the previous 3 months; or active or chronic hepatobiliary or hepatic disease or severe renal impairment; and if they were pregnant or lactating women or were participating in another clinical trial.

### Randomization and blinding

Randomization sequence was computer-generated with a 1:1 allocation using a random block size of 4. The randomized treatment assignments were sealed in opaque envelopes. Physicians, outcome assessors, and data analysts were blinded regarding the treatment allocation. XXK capsule and its placebo, as well as CDS tablet and its placebo were identical in external appearance, texture, taste and smell.

### Interventions

Patients were randomly assigned to receive active XXK capsule (100 mg per capsule containing 35 mg dioscin) and placebo CDS tablet; or CDS tablet (250 mg per tablet) and placebo XXK capsule, which were manufactured by Chengdu Di'ao Pharmaceutical Group of China. Each dose included 2 capsules and 2 tablets, 3 times per day, and the duration of treatment was 20 weeks. All other antianginal medications were proscribed except sublingual nitroglycerin as required. If any other medication or therapy was required to treat the concomitant diseases, then the name of the drug or therapy, actual dosage, dosing frequency and start/stop time should be well-documented.

Compliance with the therapeutic regimen was evaluated at each visit (week 2, 4, 6, 8 and 20) by counting the number of returned capsules and tablets.

### Outcome measures

The primary outcome measure was the proportion of patients who were angina-free, defined as patient anginal symptoms evaluated as “none” on all of dimensions of Quantification Score of Angina Pectoris (QSAP)^48^. The QSAP includes three dimensions (attack frequency, severity, and duration of angina) and comprises questions investigating patient anginal symptoms during a 2-week period (Details can be found in Supplementary Appendix 1). It was administered by two experienced clinicians from each study site at each visit.

Effectiveness analyses for the primary end-point (angina-free) were also conducted in subgroups stratified by sex, age, angina severity, concomitant disease, weekly-angina frequency, nitroglycerin use, disease duration, concomitant medication, and prior treatment.

Another primary outcome measure was the proportion of patients with normal ECG recordings. The “normal ECG” in our trial is defined as including (1) normal sinus rhythm (each P wave is followed by a QRS and P wave rate 60–100 bpm with <10% variation); (2) normal P waves (height < 2.5 mm in lead II and width < 0.11 s in lead II); (3) normal PR interval (0.12 to 0.20 s); (4) normal QRS complex (<0.12 s duration, no pathological Q waves and no evidence of left or right ventricular hypertrophy); (5) normal QT interval (0.42 s); (6) normal ST segment without any elevation or depression; (7) normal T wave; and (8) normal U wave. A standard 12-lead ECG was performed and the findings were interpreted by two ECG specialists from each study site at baseline, week 8 and week 12, respectively.

The secondary effectiveness measures included the changes from baseline in self-reported weekly angina frequency over the 20-week intervention period and average weekly nitroglycerin consumption rate at week 8 and week 20. Throughout the study, patients were asked to record the occurrence of anginal attacks and nitroglycerin use on diary cards, and the study staff at each clinical site reviewed the patient diary cards with the patient at each scheduled visit to ensure accuracy.

Other important secondary outcome measures were the changes from baseline in 5 dimensions of the SAQ^49^, and the proportions of patients with clinically significant improvement at week 8 and week 20. Clinically significant improvement was defined as a difference of at least 8 points on the physical-limitation scale, 25 points on the angina-stability scale, 20 points on the angina-frequency scale, 12 points on the treatment-satisfaction scale, and 16 points on the quality-of-life scale^50^.

Additional outcome measures involved a common TCM syndrome in patients with chronic stable angina - *Xueyu Zheng*. We evaluated the change from baseline in the *Xueyu Zheng* score and the proportion of patients who had significant syndrome improvement. The *Xueyu Zheng* score is a 6-item questionnaire that quantifies the symptoms and signs of stable angina including the angina, choking sensation in the chest, palpitation, dark purple lips, ecchymosis on the tongue, and fine-choppy pulse^47^. Each of these six dimensions was scored separately according to the judgment of the clinicians for the pathophysiological status of the patients: the first item (angina) was 0, 2, 4, 6; in second to fourth items (choking sensation in the chest, palpitation, dark purple lips) were from 0 to 3; in fifth item (ecchymosis on the tongue) was from 0 to 2; and the last item about fine-choppy pulse was 0 or 1. Details can be found in Supplementary Appendix 1, with higher scores indicating higher severity. Two experienced clinicians from each study site assessed the syndrome score at each visit. Significant syndrome improvement was defined as at least 70% reduction in the *Xueyu Zheng* score^48^.

Besides, other changes from baseline were also analyzed and reported in this paper, including changes in the time to onset of ST segment depression, time to onset of angina, and maximum ST segment depression in the first 1/3 of patients included at each site who received ETT, as well as changes in blood lipid at week 8.

### Safety and tolerability assessments

Throughout the entire intervention period, all AEs spontaneously reported by the patient or observed by the investigator were recorded, regardless of whether or not they were considered to be drug-related. The investigators reviewed all the AEs, using a standard AE case report form to collect information and then assessed if the AE was related to the drug. In addition, routine clinical laboratory tests including haematology and blood chemistry were performed at baseline and the end of the intervention period.

### Statistical analysis

Since this trial adopts an adaptive design (under the protocol), we initially estimated a sample size of 576 subjects, which would provide approximately 80% power in detecting a relative increase of 10% in the proportion of angina-free patients (using a 2-sided test at α = 0.05) in the XXK group as compared with the control group, assuming an increase of 20% in the control group. An interim analysis with the use of O'Brien-Fleming stopping boundaries was performed after 288 patients were recruited. On the advice of the Data and Safety Monitoring Board and on the basis of lower-than-expected proportions of angina-free patients, the sample size was recalculated and expanded to enroll 736 patients (Full details can be found in the study protocol in Supplementary Appendix 1).

Statistical analyses were performed in the intent-to-treat (ITT) population^51^, which was defined as all randomized patients who received at least one dose of study drug. Missing data was handled using last observation carried forward (LOCF) imputation technique^52^. Continuous variables were reported as mean and standard deviation (SD) and categorical variables as frequencies and percentages with 95% confidence intervals (CIs). Statistical differences between groups were analyzed using t test for quantitative data and Chi-sqare test for categorical data. All reported P values were two-sided; and a P value less than 0.05 was considered to be statistically significant except that from the primary effectiveness analyses, in which Bonferroni's multiple comparison test was applied for twice and P < 0.025 was considered significant. All calculations were performed using SAS 9.1.

## Author Contributions

As the guarantor, Y.Y.W. conceived the study. Y.N.Y., Z.W. and J.L. initially drafted the paper with subsequent contributions from all authors. X.L.L., X.Y.Y., B.D., D.H.W., X.F.W. and S.R.L. enrolled participants and collected the data under the supervision of S.Y.H., G.X.L., J.X. and Z.M.L. B.W.C. and L.Y.W. cleaned and analyzed the data. S.S.L. coordinated the study. Q.G.C. and C.T.S. monitored the conduct of the study and reviewed the safety and effectiveness data. All authors reviewed the manuscript.

## Supplementary Material

Supplementary Information Research checklist and Supplementary Appendix 1

## Figures and Tables

**Table 1:** Baseline characteristics of study participants*

Characteristics	XXK group (N = 366)	CDS group (N = 367)	P value
**Sex — no. (%)**			0.6696
**Male**	113(30.87)	108(29.43)	
**Female**	253(69.13)	259(70.57)	
**Age (yr)**	58.05 ± 7.75	58.22 ± 7.36	0.7597
**Prior treatment for angina—No. (%)**			0.3004
**Yes**	183(50.00)	197(53.68)	
**No**	183(50.00)	170(46.32)	
**Concomitant disease—No. (%)**†			0.5979
**Yes**	81(22.13)	87(23.71)	
**No**	285(77.87)	280(76.29)	
**Mean pulse rate (bpm)**£	70.20 ± 8.65	71.62 ± 8.77	0.0285‡
**Systolic pressure (mmHg)**	124.34 ± 8.77	125.56 ± 8.65	0.0573
**Diastolic pressure (mmHg)**	76.64 ± 6.39	77.05 ± 6.86	0.3969
**Weekly angina frequency**	6.95 ± 3.99	6.94 ± 3.78	0.9549
**CCS angina class—No. (%)**§			0.8659
**I**	104(28.42)	102(27.79)	
**II**	247(67.49)	250(68.12)	
**III**	15(4.10)	15(4.09)	
**Weekly nitroglycerin use**	1.81 ± 3.08	1.75 ± 2.9	0.9816
**SAQ score**§			
**Angina frequency**	54.62 ± 19.70	54.39 ± 19.09	0.8722
**Physical limitation**	68.90 ± 16.35	69.80 ± 16.52	0.4579
**Angina stability**	41.67 ± 18.47	42.78 ± 17.60	0.4041
**Treatment satisfaction**	49.47 ± 17.57	49.91 ± 16.71	0.7252
**Quality of life**	40.57 ± 17.41	41.42 ± 16.73	0.5040
***Xueyu Zheng* score**§	7.09(1.79)	7.06(1.77)	0.8184
**ECG—No. (%)**¶			1.0000
**Normal**	2(0.55)	2(0.55)	
**Abnormal**	362(99.45)	364(99.45)	
**Exercise Tolerance Testing**¤			
**Time to onset of ST segment depression(s)**	292.21 ± 279.53	242.03 ± 230.97	0.1365
**Time to onset of angina(s)**∮	604.50 ± 351.01	434.22 ± 322.79	0.0560
**Maximum ST segment depression(mv)**	0.19 ± 0.16	0.22 ± 0.33	0.3919

*Plus–minus values are means ± SD unless otherwise noted.

†The “concomitant disease” mainly included diabetes mellitus, hypertension or hyperlipidemia.

£Data were missing for one patient in the XXK group and three patients in the CDS group.

‡The mean heart rate in the XXK group was lower than that in the CDS group, P < 0.05.

§“CCS” referred to Canadian Cardiovascular Society. “SAQ” referred to Seattle Angina Questionnaire.

¶“ECG” referred to electrocardiogram and the data were missing for two patients in the XXK group and one patient in the CDS group.

¤Exercise Tolerance Testing (ETT) was administrated in 111 and110 patients in the XXK and CDS groups, respectively.

∮During the exercise testing, 34 and 27 patients experienced an onset of angina in the XXK and CDS groups, respectively.
