# Supplementary material for: Comparative Effectiveness of Di'ao Xin Xue Kang Capsule and Compound Danshen Tablet in Patients With Symptomatic Chronic Stable Angina
Source: Sci Rep. 2014 Nov 14;4:7058. doi: 10.1038/srep07058 (PMC4231340; doi:10.1038/srep07058)
Supplement: Supplementary Information — Research checklist and Supplementary Appendix 1 [file srep07058-s1.pdf]

Comparative Effectiveness of Di'ao Xin Xue Kang Capsules and Compound Danshen Tablet in Patients With Symptomatic Chronic Stable Angina

Yanan Yu[1], Siyuan Hu[2], Guoxin Li [3], Jie Xue [4], Zhuoming Li[5], Xiangling Liu[2], Xiyan Yang[2], Bo Dong[3], Donghai Wang[3], Xiaofeng Wang[4], Shurong Liu[5], Jun Liu[1], Bingwei Chen[6], Liying Wang[1], Songshan Liu[7], Qiguang Chen[6], Chunti Shen[8], Zhong Wang[1 *], Yongyan Wang [1*]

[1]Institute of Basic Research in Clinical Medicine, China Academy of Chinese Medical Sciences, No. 16 Nanxiaojie, Dongzhimen nei, Beijing, 100700, China

[2]First Teaching Hospital of Tianjin University of Traditional Chinese Medicine, AnShan Xi Road 314 , Nankai District , 300193, Tianjin, China

[3]The Second Hospital Affiliated to Liaoning University of TCM, HuangHe North Road 60, Huanggu District, Shenyang 110034, Liaoning, China

[4]The TCM Hospital of Xinjiang Uygur Autonomous Region, HuangHe Road 116, Wulumuqi 830099, Xinjiang, China

[5]The Jilin Provincial Hospital of Integrated TCM and Western Medicine, Gongnong Da Road 1745, Chaoyang District, Changchun 130021, Jilin, China

[6]School of Public Health, Southeast University, Dijia Qiao 87, Nanjing 210009, Jiangsu, China

[7]The Affiliated Hospital of Chengdu University of Traditional Chinese Medicine, No.37 Twelve Road, Chengdu 610075, Sichuan, China

[8]Changzhou TCM Hospital, Heping North Road, Tianing District, Changzhou 213004, Jiangsu, China

* **Corresponding Authors:**

1. Zhong Wang

Institute of Basic Research in Clinical Medicine, China Academy of Chinese Medical Sciences, No.16 Nanxiaojie, Dongzhimennei, Beijing, 100700, China. Fax: 86-10-84046033; Tel: 86-10-64014411-3308; Email: zhonw@vip.sina.com

2. Yongyan Wang

Institute of Basic Research in Clinical Medicine, China Academy of Chinese Medical Sciences, No.16 Nanxiaojie, Dongzhimennei, Beijing, 100700, China. Fax: 86-10-84046033; Tel: 86-10-84046033, E-mail: wangyongyan2010@sina.cn

CONSORT 2010 checklist of information to include when reporting a randomised trial*

Section/Topic	Item No	Checklist item	Reported on page No
**Title and abstract**			
	1a	Identification as a randomised trial in the title	1
	1b	Structured summary of trial design, methods, results, and conclusions (for specific guidance see CONSORT for abstracts)	3
**Introduction**			
Background and objectives	2a	Scientific background and explanation of rationale	4-6
	2b	Specific objectives or hypotheses	6
**Methods**			
Trial design	3a	Description of trial design (such as parallel, factorial) including allocation ratio	13
	3b	Important changes to methods after trial commencement (such as eligibility criteria), with reasons	N/A
Participants	4a	Eligibility criteria for participants	13-14
	4b	Settings and locations where the data were collected	13
Interventions	5	The interventions for each group with sufficient details to allow replication, including how and when they were actually administered	14-15
Outcomes	6a	Completely defined pre-specified primary and secondary outcome measures, including how and when they were assessed	15-17
	6b	Any changes to trial outcomes after the trial commenced, with reasons	N/A
Sample size	7a	How sample size was determined	18
	7b	When applicable, explanation of any interim analyses and stopping guidelines	18
Randomisation:			
Sequence generation	8a	Method used to generate the random allocation sequence	14
	8b	Type of randomisation; details of any restriction (such as blocking and block size)	14
Allocation concealment mechanism	9	Mechanism used to implement the random allocation sequence (such as sequentially numbered containers), describing any steps taken to conceal the sequence until interventions were assigned	14
Implementation	10	Who generated the random allocation sequence, who enrolled participants, and who assigned participants to interventions	14-15

Blinding	11a	If done, who was blinded after assignment to interventions (for example, participants, care providers, those assessing outcomes) and how	14
	11b	If relevant, description of the similarity of interventions	14
Statistical methods	12a	Statistical methods used to compare groups for primary and secondary outcomes	18
	12b	Methods for additional analyses, such as subgroup analyses and adjusted analyses	N/A
**Results**			
Participant flow (a diagram is strongly recommended)	13a	For each group, the numbers of participants who were randomly assigned, received intended treatment, and were analysed for the primary outcome	6
	13b	For each group, losses and exclusions after randomisation, together with reasons	Figure 1
Recruitment	14a	Dates defining the periods of recruitment and follow-up	6
	14b	Why the trial ended or was stopped	N/A
Baseline data	15	A table showing baseline demographic and clinical characteristics for each group	Table 1
Numbers analysed	16	For each group, number of participants (denominator) included in each analysis and whether the analysis was by original assigned groups	6
Outcomes and estimation	17a	For each primary and secondary outcome, results for each group, and the estimated effect size and its precision (such as 95% confidence interval)	7-9
	17b	For binary outcomes, presentation of both absolute and relative effect sizes is recommended	7-9
Ancillary analyses	18	Results of any other analyses performed, including subgroup analyses and adjusted analyses, distinguishing pre-specified from exploratory	7<br>Figure 3
Harms	19	All important harms or unintended effects in each group (for specific guidance see CONSORT for harms)	9
**Discussion**			
Limitations	20	Trial limitations, addressing sources of potential bias, imprecision, and, if relevant, multiplicity of analyses	12
Generalisability	21	Generalisability (external validity, applicability) of the trial findings	9-12
Interpretation	22	Interpretation consistent with results, balancing benefits and harms, and considering other relevant evidence	9-12
**Other information**			
Registration	23	Registration number and name of trial registry	13
Protocol	24	Where the full trial protocol can be accessed, if available	Supplementary Appendix 1
Funding	25	Sources of funding and other support (such as supply of drugs), role of funders	26

*We strongly recommend reading this statement in conjunction with the CONSORT 2010 Explanation and Elaboration for important clarifications on all the items. If relevant, we also recommend reading CONSORT extensions for cluster randomised trials, non-inferiority and equivalence trials, non-pharmacological treatments, herbal interventions, and pragmatic trials. Additional extensions are forthcoming: for those and for up to date references relevant to this checklist, see www.consort-statement.org.

Comparative Effectiveness of Di'ao Xin Xue Kang Capsules and Compound Danshen Tablet in Patients With Symptomatic Chronic Stable Angina

Yanan Yu[1], Siyuan Hu[2], Guoxin Li [3], Jie Xue [4], Zhuoming Li[5], Xiangling Liu[2], Xiyan Yang[2], Bo Dong[3], Donghai Wang[3], Xiaofeng Wang[4], Shurong Liu[5], Jun Liu[1], Bingwei Chen[6], Liying Wang[1], Songshan Liu[7], Qiguang Chen[6], Chunti Shen[8], Zhong Wang[1 *], Yongyan Wang [1*]

[1]Institute of Basic Research in Clinical Medicine, China Academy of Chinese Medical Sciences, No. 16 Nanxiaojie, Dongzhimen nei, Beijing, 100700, China

[2]First Teaching Hospital of Tianjin University of Traditional Chinese Medicine, AnShan Xi Road 314 , Nankai District , 300193, Tianjin, China

[3]The Second Hospital Affiliated to Liaoning University of TCM, HuangHe North Road 60, Huanggu District, Shenyang 110034, Liaoning, China

[4]The TCM Hospital of Xinjiang Uygur Autonomous Region, HuangHe Road 116, Wulumuqi 830099, Xinjiang, China

[5]The Jilin Provincial Hospital of Integrated TCM and Western Medicine, Gongnong Da Road 1745, Chaoyang District, Changchun 130021, Jilin, China

[6]School of Public Health, Southeast University, Dijia Qiao 87, Nanjing 210009, Jiangsu, China

[7]The Affiliated Hospital of Chengdu University of Traditional Chinese Medicine, No.37 Twelve Road, Chengdu 610075, Sichuan, China

[8]Changzhou TCM Hospital, Heping North Road, Tianing District, Changzhou 213004,

Jiangsu, China

* **Corresponding Authors:**

1. Zhong Wang

Institute of Basic Research in Clinical Medicine, China Academy of Chinese Medical Sciences, No.16 Nanxiaojie, Dongzhimennei, Beijing, 100700, China. Fax: 86-10-84046033; Tel: 86-10-64014411-3308; Email: zhonw@vip.sina.com

2. Yongyan Wang

Institute of Basic Research in Clinical Medicine, China Academy of Chinese Medical Sciences, No.16 Nanxiaojie, Dongzhimennei, Beijing, 100700, China. Fax: 86-10-84046033; Tel: 86-10-84046033, E-mail: wangyongyan2010@sina.cn

Protocol

Efficacy and safety of Xinxuekang capsule in the treatment of angina pectoris associated with coronary heart disease (blood stasis syndrome): A multi-center, randomized, parallel-controlled, double-blind, adaptive clinical trial (superiority design)

Sponsored by: This study was supported by the National Natural Science Foundation of China (30973966) and National Science and Technology Major Projects (2008ZX09312-012).

Complications Scientific Committee Chair: Prof. Yong-Yan Wang

Protocol Co-Chairs: Prof. Zhong Wang, Ph.D

Protocol Vice Chairs: Prof. Si-Yuan Hu, Ph.D.; Prof. Guo-Xin Li, Ph.D.; Prof. Jie Xue, Ph.D.; Prof. Zhuo-Ming Li

Clinical Trial Specialists: Jun Liu, Ph.D; Yanan Yu

PARTICIPATING SITES

First Teaching Hospital of Tianjin University of Traditional Chinese Medicine, AnShan Xi Road 314 , Nankai District 300193, Tianjin, China

The Second Hospital Affiliated to Liaoning University of TCM, HuangHe North Road 60,

Huanggu District, Shenyang 110034, Liaoning, China

The TCM Hospital of Xinjiang Uygur Autonomous Region, HuangHe Road 116, Wulumuqi 830099, Xinjiang, China

The Jilin Provincial Hospital of Integrated TCM and Western Medicine, Gongnong Da Road 1745, Chaoyang District, Changchun 130021, Jilin, China

PROTOCOL TEAM ROSTER

Protocol Co-Chairs

Prof. Yong-Yan Wang

Co-Director, Institute of Basic Research in Clinical Medicine, China Academy of Chinese Medical Sciences, No. 16 Nanxiaojie, Dongzhimen nei, Beijing, 100700, China

Tel: 86-10-84046033, E-mail: wangyongyan2010@sina.cn

Prof. Zhong Wang, Ph. D

Institute of Basic Research in Clinical Medicine, China Academy of Chinese Medical Sciences, No. 16 Nanxiaojie, Dongzhimen nei, Beijing, 100700, China

Tel: 86-10-64014411-3308, E-mail: zhonw@vip.sina.com

Protocol Vice Chairs

Prof. Si-Yuan Hu, Ph.D.

First Teaching Hospital of Tianjin University of Traditional Chinese Medicine
AnShan Xi Road 314 , Nankai District , 300193, Tianjin, China

E-mail: husiyuan1963@sina.com

Prof. Guo-Xin Li, Ph.D.

The Second Hospital Affiliated to Liaoning University of TCM

HuangHe North Road 60, Huanggu District, Shenyang 110034, Liaoning, China

E-mail:syyljdlgx024@126.com

Prof. Jie Xue, Ph.D.

The TCM Hospital of Xinjiang Uygur Autonomous Region

HuangHe Road 116, Wulumuqi 830099, Xinjiang, China

E-mail: kjxuejie@163.com

Prof. Zhuo-Ming Li

The Jilin Provincial Hospital of Integrated TCM and Western Medicine

Gongnong Da Road 1745, Chaoyang District, Changchun 130021, Jilin, China

Email:jdlzm16@yahoo.com.cn

Clinical Trial Specialists

Jun Liu, Ph. D

Institute of Basic Research in Clinical Medicine, China Academy of Chinese Medical Sciences,

No. 16 Nanxiaojie, Dongzhimen nei, Beijing, 100700, China

E-mail: franlj1104@yahoo.com.cn

Yanan Yu

Institute of Basic Research in Clinical Medicine, China Academy of Chinese Medical Sciences,

No. 16 Nanxiaojie, Dongzhimen nei, Beijing, 100700, China

E-mail: pumpkinnaicha@163.com

Tianjin Investigators

Prof. Si-Yuan Hu, Ph.D.

Email: husiyuan1963@sina.com

Xi-Yan Yang

Email: Yangxiyan888@yahoo.com.cn

Xiang-Ling Liu

Email: liuxiangling1999@163.com

Liaoning Investigators

Prof. Guo-Xin Li, Ph.D.

Email: syyljdlgx024@126.com

Prof. Bo Dong

Email: 18940158816@163.com

Dong-Hai Wang

Email: donghai1977@126.com

Xinjiang Investigators

Prof. Jie Xue, Ph.D.

Email: kjxuejie@163.com

Prof. Xiao-Feng Wang

Email: wxf87112008@163.com

Changchun Investigators

Prof. Zhuo-Ming Li

Email:qawbs@sohu.com

Prof. Shu-Rong Liu

Email: shurongliu@sohu.com

Protocol Statisticians

Prof. Bi-Wei Chen, Ph.D.

School of Public Health, Southeast University

Dijia Qiao 87, Nanjing,210009, Jiangsu,China

E-mail: drchenbw@126.com

Prof. Wei Lu, Ph.D.

Department of Pharmaceutics, School of Pharmaceutical Science, Peking University Health Science Center, Xueyuan Rd, HaiDian District, Beijing 100083, China

E-mail: luwei_pk@bjmu.edu.cn

Li-Ying Wang, Ph.D.

Institute of Basic Research in Clinical Medicine, China Academy of Chinese Medical Sciences

Dongzhimennei Nanxiaojie 16#, Beijing 100700, China

E-mail: coldmoon_ly@163.com

Data monitoring committee (DMC)

Prof. Chun-Ti Shen

Changzhou TCM Hospital, Heping North Road, Tianing District, Changzhou 213004, Jiangsu, China

Email:czsct@163.com

Prof. Qi-Guang Chen

School of Public Health, Southeast University

Dijia Qiao 87, Nanjing,210009, Jiangsu,China

Email: chwa2000@sohu.com

Prof. Zhi-Wei Jing, Ph.D.

Institute of Basic Research in Clinical Medicine, China Academy of Chinese Medical Sciences

Dongzhimennei Nanxiaojie 16#, Beijing 100700, China

Email: drjzw@163.com

Clinical Study Coordinators

Pro. Song-Shan Liu

The Affiliated Hospital of Chengdu University of Traditional Chinese Medicine,

No.37 Twelve Road, Chengdu, 610075, Sichuan, China

E-mail: pine009@163.com

Li-Ya Su

Institute of Basic Research in Clinical Medicine, China Academy of Chinese Medical Sciences,

No. 16 Nanxiaojie, Dongzhimen nei, Beijing, 100700, China

Tel: 86-0-15101016441; E-mail: suliya5641@163.com

Hong-Li Wu

Institute of Basic Research in Clinical Medicine, China Academy of Chinese Medical Sciences,

No. 16 Nanxiaojie, Dongzhimen nei, Beijing, 100700, China

Tel: 86-0-13716165325; E-mail: thisyear2006@126.com

PROTOCOL SIGNATURE PAGE

Efficacy and safety of Xinxuekang capsule in the treatment of angina pectoris associated with coronary heart disease (blood stasis syndrome): A multi-center, randomized, parallel-controlled, double-blind, adaptive clinical trial (superiority design)

Protocol Final Version Approval

This protocol has been verified and approved by:

Guo-Xin Li Si-Yuan Hu Jie Xue Zhuo-Ming Li 2008-12-15

Principal investigators Date

Chun-Ti Shen Qi-Guang Chen Zhi-Wei Jing 2008-12-15

Data monitoring committee (DMC) Date

Song-Shan Liu Li-Ya Su Hong-Li Wu 2008-12-15

Clinical Monitor Date

TABLE OF CONTENTS

PARTICIPATING SITES......3
PROTOCOL TEAM ROSTER......4
PROTOCOL SIGNATURE PAGE......9
GLOSSARY......12
PROTOCOL SYNOPSIS......14
1. BACKGROUND INFORMATION......16
2. OBJECTIVES......16
3. STUDY DESIGN......16
3.1 Superiority design......17
3.2 Sample size......17
Initial estimation of sample size......17
Sample size re-estimation......17
3.3 Randomization and case assignment......18
3.4 Selection of control group......19
3.5 Blinding......19
3.6 Data Monitoring Committee (DMC) and Implementation & Decision-making Committee......20
3.7 Interim analysis......20
4. SELECTION AND WITHDRAWAL OF STUDY PARTICIPANTS......20
4.1 WM (Western Medicine) and TCM diagnostic criteria of angina pectoris......20
4.1.1 WM diagnostic criteria of angina pectoris......21
4.1.2 TCM diagnostic criteria of angina pectoris (blood stasis syndrome) 21
4.2 Grading of angina pectoris......21
4.3 Quantification score of angina pectoris......22
4.4 Quantification score of TCM syndrome (phenotypic group)......23
4.5 Eligibility criteria......24
4.5.1 Inclusion criteria......25
4.5.2 Exclusion criteria......25
4.5.3 Withdrawal/dropout criteria......25
4.5.4 Criteria for study termination......26
4.5.5 Criteria for subject discontinuation......26
5 TEARTMENT REGIMEN......27
5.1 Origins of study drugs......27
5.2 Packaging of study drugs......27
5.3 Accountability procedures for study drugs......28
5.4 Study treatment......28
6 OBSERVATION INFORMATION......29
6.1 Safety parameters......29
6.2 Efficacy parameters......29
7 EFFICACY EVALUATION......31
7.1 Criteria for overall efficacy evaluation......31
7.2 Evaluation of angina changes......31
7.3 Evaluation of syndrome changes......32

7.4 Evaluation of ECG changes 32
7.5 Evaluation of changes in symptoms or signs 32
7.6 Use of nitroglycerin 33
8 ADVERSE EVENTS 33
8.1 Background information about drug-related safety issues 33
8.2 Monitoring and recording of adverse events 33
8.2.1 Definition 33
8.2.2 Recording 34
8.3 Severity of An AE 34
8.4 Relationship to study drugs 34
8.5 Handling of SAEs 35
8.6 Opening and handling of emergency envelops 35
8.7 Follow-up of unresolved AEs 35
9 SAFETY EVALUATION 35
10 DATA MANAGEMENT 36
10.1 Collection and fulfillment of CRF 36
10.2 Data collection and management 36
10.3 Data Lock 36
11 STATISTICAL CONSIDERATIONS AND ANALYTICAL PLAN 36
11.1 Selection of data sets 36
11.2 Dynamic statistical plan 37
12 QUALITY CONTROL AND QUALITY ASSURANCE 38
12.1 Laboratory quality control 38
12.2 Investigator training 38
12.3 Compliance enhancement 39
12.4 Study monitoring 39
12.5 Multi-center study coordination committee 39
13 ETHICAL ASPECTS 40
14 STUDY DOCUMENTATION, CRFS AND RECORD KEEPING 40
14.1 Investigator's files/retention of documents 40
14.2 Source documents and background data 41
14.3 Audits and inspections 41
14.4 Case Report Forms 41
15 CLINICAL TRIAL FLOWCHART 42
16 PUBLICATION POLICY 43
17 REFERENCES 43
18 CONDITIONS FOR MODIFYING THE PROTOCOL 44

GLOSSARY

ADR adverse drug reaction

AE adverse event

ANCOVA analysis of covariance

ANOVA analysis of variance

BP blood pressure

CDS Compound Danshen

CHD coronary heart disease

CMH Cochran-Mantel-Haenszel

CSC clinical study coordinator

CRF case report form

DMC Data Monitoring Committee

DQF data queries form

ECG electrocardiogram

FAS full analysis set

HDL high density lipoprotein

LDL low density lipoprotein

LOCF last observation carried forward

MDS multidimensional scaling analysis

NONMEM nonlinear mixed effect model

PPS per protocol set

SAQ Seattle Angina Questionnaire

SAE serious adverse event

SAP stable angina pectoris

SDA similarity degree analysis

SFDA State Food and Drug Administration of China

SOP standard operating procedure

SS safety analysis set

TCM traditional Chinese medicine

WM western medicine

XXK Xinxuekang

SUMMARY OF CHANGES

Summary of Changes for ChiCTR-TRC-09000332,Efficacy and Safety of Xinxuekang Capsule in the Treatment of Angina Pectoris Associated with Coronary Heart Disease (Blood Stasis Syndrome): A Multi-Center, Randomized, Parallel-Controlled, Double-Blind, Adaptive Clinical Trial

All changes in this version appear in boldface and italic type. Major changes include the following:

1) **In Section 3.2, A total of 576 study participants with angina pectoris was planned initially. After the interim analysis, the sample size was re-estimated to be 736.**
2) **In Section 3.3, After the sample size was expanded to 736 patients, the final case assignment in 4 participating sites aslo changed(184 patients in each site; 92 in the trial group and 92 in the control group).**

PROTOCOL SYNOPSIS

TITLE:

Efficacy and safety of Xinxuekang capsule in the treatment of angina pectoris associated with coronary heart disease (blood stasis syndrome): A multi-center, randomized, parallel-controlled, double-blind, adaptive clinical trial (superiority design)

DESIGN:

A multi-center, randomized, parallel-controlled, double-blind, adaptive clinical trial (superiority design)

NUMBER OF SITES:

4 sites

SAMPLE SIZE:

A total of 576 study participants with angina pectoris was planned initially. ***After the interim analysis, the sample size was re-estimated to be 736.***

POPULATION:

Patients aged 40 to 70 years who suffered from stable effort angina, CCS angina class Ⅰ, Ⅱ or Ⅲ with an attack frequency equal to or greater than 2 episodes per week were eligible for inclusion in the study.

RANDOMIZATION：

Randomization to XXK capsule or CDS tablet will be in a 1:1 ratio

REGIMEN:

Trial group: XXK capsule + placebo of CDS tablet, oral, 2 capsules + 2 tablets a time, 3 times a day;

Control group: CDS tablet + placebo of XXK capsule, oral, 2 tablets + 2 capsules a time, 3 times a day.

TREATMENT DURATION:

20 weeks treatment.

OUTCOME MEASURES:

Efficacy:

The primary efficacy endpoint of this study is:

- Proportions of patients who were angina-free.
- Proportion of effective electrocardiogram (ECG) improvement.

Secondary efficacy endpoints are:

- Change from baseline in weekly average frequency of self-reported angina episode over the 20-weeks treatment phase.
- Weekly average nitroglycerin consumption rate at week 8 and week 20.
- Change from baseline in 5 dimensions of the Seattle Angina Questionnaire (SAQ) and the proportions of patients who were clinical significance at week 8 and week 20.
- Change from baseline in the blood stasis syndrome and the proportion of patients who had significant syndrome improvement.
- Change from baseline in ECG exercise test (the first 1/3 of patients included at each site)performed at 0 and 8 weeks.
- Change from baseline in serum lipid: total cholesterol, triglyceride, HDL cholesterol, and LDL cholesterol, measured at 0 and 8 weeks.
- Related signs: heart rate, rhythm, murmur, and BP, recorded before and after Treatment.
- Accompanying symptoms after treatment: fatigue, shortness of breath, aversion to cold, cold extremities and etc. (recorded as “yes” or “no”).

Safety:

- Vital signs;
- Blood, urine and stool routine tests;
- Liver function (ALT), kidney function (BUN and Cr);
- Any AE that occurs throughout the treatment period.

1. BACKGROUND INFORMATION

Xinxuekang (XXK), an oral capsule, is a pure traditional Chinese medicine (TCM) preparation manufactured by modern scientific techniques. It is indicated for the treatment of angina pectoris associated with coronary heart disease (blood stasis syndrome or “Xueyu zheng”), which has the functions of promoting blood circulation, removing blood stasis, promote qi circulation and relieving pain. It has been approved by the State Food and Drug Administration of China (SFDA) and widely used to treat angina in Chinese medical practice for many years.

Pre-clinical studies of XXK capsule mainly include pharmacokinetic, pharmacodynamic and toxicological (acute and chronic toxicities) studies, which are summarized in separate brochures.

2. OBJECTIVES

To investigate the clinical pharmacological characteristics of XXK capsule in the treatment of angina associated with coronary heart disease (blood stasis syndrome).

- To evaluate the efficacy of XXK capsule versus Compound Danshen (CDS) tablets in the treatment of angina associated with coronary heart disease (blood stasis syndrome) by analyzing the proportions of patients who were angina-free, changes in the frequency, intensity and duration of angina, use of nitroglycerin, electrocardiogram (ECG) readings, changes in the SAQ and TCM syndrome transformation;
- To evaluate the efficacy of XXK capsule versus CDS tablets in the treatment of angina associated with coronary heart disease (blood stasis syndrome) by analyzing changes in blood coagulation, lipid and hemorheological parameters;
- To evaluate the safety of XXK capsule in the treatment of angina associated with coronary heart disease (blood stasis syndrome) by analyzing changes in blood (RBC, Hb, PLT, WBC, N, and L), urine and stool routine tests, liver (ALT) and kidney (BUN and Cr) functions before and after treatment, the frequency and incidence of adverse events (AEs) during treatment as well as their relationship to the study drug.

3. STUDY DESIGN

3.1 Superiority design

An adaptive design is applied in this clinical trial, which utilizes accumulating data to modify aspects of the study as it continues, without undermining the validity and integrity of the trial. “Validity” refers to deducing correct statistical analysis without comprising the bias that might include protecting the Type I error; and “integrity” refers to preplanning, as much as possible, based on intended adaptations, maintaining confidentiality of data, and providing convincing results to a broader scientific community.

This study aims to evaluate the efficacy and safety of XXK capsule versus CDS tablets in the treatment of angina associated with coronary heart disease (blood stasis syndrome).

Four qualified study sites will be recruited to conduct the trial.

Due to the adaptive design, one interim analyses is planned. If the superiority of the trial group is demonstrated at the time of interim analysis, then the trial should be terminated, which is also satisfies the ethical considerations.

3.2 Sample size

Initial estimation of sample size

Since this trial adopts an adaptive design, we initially estimated the sample size based on one of the primary efficacy endpoints, i.e. the proportions of patients who were angina-free according to findings from other and previous studies[1-2]. We assume that the effective rate in the proportions of patients who were angina-free of the trial group was 30%, while that of the control group was 20%. We chose a significance level of 0.05 and a target test power of 0.80. The trial is planned to enroll 465 patients. Considering the dropout rate and stratified block based on the site, the sample size is enlarged to 576 and one planned interim analyses by a Data Monitoring Committee (DMC) will be performed after 288 patients enrolled. Enrollment will be suspended if XXK capsule is found to be significantly more efficacious than the CDS tablets and the O’Brien–Fleming spending function according to the method of Lan and Demets will be applied.

Sample size re-estimation

In the interim analysis (288 patients enrolled), according to the previous accumulative data in this trial, the proportions of patients who were angina-free

between the two groups can not reach the statistical power. The proportions in angina-free of the trial group was 29.17%, while that of the control group was 20.99%. Therefore, the sample size was expanded to enroll 736 patients considering a low dropout rate.

3.3 Randomization and case assignment

For the allocation of participants, a computer-generated list of random numbers was used. Randomization sequence was created using SAS 9.1 statistical software and was stratified by study site with a 1:1 allocation using random block sizes of 2, 4, and 4. Study participants will be serially numbered as 001-576 and randomly assigned to receive treatment with XXK capsule or CDS tablets (288 in the trial group and 288 in the control group).

Case assignment in 4 participating sites

Sites	Trial group	Control group	n
The First Hospital Affiliated to Tianjin University of TCM	72	72	144
The Second Hospital Affiliated to Liaoning University of TCM	72	72	144
The TCM Hospital of Xinjiang Uygur Autonomous Region	72	72	144
The Jilin Provincial Hospital of Integrated TCM and Western Medicine	72	72	144

Changes:

After the interim analysis, the sample size was re-estimated and expanded to enroll 736 patients.Therefore,160 cases (numbered as 577-736)were added and randomly assigned to receive XXK capsule or CDS tablets (80 in the trial group and 80 in the control group) and the final case assignment in 4 participating sites was:

Sites	Trial group	Control group	n

The First Hospital Affiliated to Tianjin University of TCM	***92***	***92***	***184***
The Second Hospital Affiliated to Liaoning University of TCM	***92***	***92***	***184***
The TCM Hospital of Xinjiang Uygur Autonomous Region	***92***	***92***	***184***
The Jilin Provincial Hospital of Integrated TCM and Western Medicine	***92***	***92***	***184***

3.4 Selection of control group

Based on the comparability principle, Compound Danshen (CDS) tablet is selected as the control group in the trial, whose indications and functions are similar to those of XXK capsule.

3.5 Blinding

Since the dosage forms of XXK capsule and CDS tablet are different, dummy preparations in both capsule and tablet forms are prepared to allow for double-blind and double-dummy comparison.

Blinding is conducted in two steps in this trial, i.e. treatment is randomly assigned to patients in the first step, and then A or B is randomly assigned to the treatment in the second step. The randomization lists of the two blinding steps are kept in sealed envelops in duplicate, one stored at the clinical study office of China Academy of Chinese Medical Sciences and the other kept by the sponsor.

Two-step unblinding will be performed after all patients have completed the trial. Step 1: after all data is locked, personnel who keeps the sealed envelop will assign A or B to either of the treatments for the conduct of statistical analysis and production of statistical reports. Step 2: after the statistical analysis is finished and the clinical trial report is prepared, the corresponding treatment that A or B refers to will be unblinded.

An emergency envelope will also be provided along with each test drug in its package, which should only be opened in case of emergency unblinding.

A patient's treatment assignment should only be unblinded when knowledge of the treatment is essential for the further management of the patient.

If the identity of the test medication is necessary for patient management (in the case of a serious complication or a serious adverse event), emergency unblinding is permitted. Investigator, project manager, and clinical study coordinators should participate in the process of emergency unblinding; when, where and why the unblinding occurs as well as each personnel's signature should also be documented. The principal investigators should make every attempt to contact the sponsor after blinding.

At the time of emergency unblinding, it is also necessary to ensure that adequate procedures are in place to ensure the integrity of the data.

3.6 Data Monitoring Committee (DMC) and Implementation & Decision-making Committee

To ensure the validity and integrity of the trial, two committees are established by the multi-center study coordination committee, i.e. Data Monitoring Committee (DMC) and Implementation & Decision-making Committee. DMC is responsible for ensuring the integrity and accuracy of data collection; and the latter (consists of leaders of each site and principal investigators) is responsible for monitoring the implementation of the trial and making important decisions related with the trial process.

3.7 Interim analysis

One interim analyses will be conducted under blinding conditions after 1/2 of the total number of cases have completed the trial. The statistical results of interim analysis will be reported to the Data and Safety Monitoring Board and Implementation & Decision-making Committee, which will decide the subsequent arrangement and calculate the needed number of cases in the next stage of trial. If one group of treatment is terminated after the interim analysis, patients in this group may choose to crossover to continue with the trial or discontinue treatment and only report their safety information (considered as dropout cases).

4. SELECTION AND WITHDRAWAL OF STUDY PARTICIPANTS

4.1 WM (Western Medicine) and TCM diagnostic criteria of angina pectoris

According to the "Guiding principle of clinical research on novel TCM preparations in the

treatment of angina associated with coronary heart disease"[3], and "Nomenclature and criteria for diagnosis of ischemic heart disease" reported by the Joint International Society and Federation of Cardiology/World Health Organization task force on standardization of clinical nomenclature[4], the diagnostic criteria of angina pectoris are developed as follows:

4.1.1 WM diagnostic criteria of angina pectoris

Angina of effort: Effort angina is characterized by transient episodes of chest pain precipitated by exercise or by other situations resulting in an increased myocardial oxygen demand. The pain usually disappears rapidly with rest or with sublingual nitroglycerin. The stable effort angina has 1 month's duration or more.

4.1.2 TCM diagnostic criteria of angina pectoris (blood stasis syndrome)[3]

Angina pectoris (blood stasis syndrome) is manifested as fixed and stabbing or gripping chest pain, which radiates to shoulder, back or the medial side of the arm, choking sensation in the chest, palpitation, dark purple lips and tongue, thin and choppy pulse.

- Main symptom: chest pain (stabbing, gripping, fixed, and radiating to shoulder, back or the medial side of the arm);
- Accompanying symptoms: chest distress, palpitation, and dark purple lips;
- Tongue and pulse: dark purple tongue, thin and choppy pulse.

If the main symptom and at least 2 accompanying symptoms are observed, along with the manifestations of tongue and pulse, the diagnosis of angina (blood stasis syndrome) could be established.

The six phenotypes of angina (blood stasis syndrome), including chest pain, choking sensation in the chest, palpitation, dark purple lips, ecchymosis on the tongue and fine-choppy pulse constitutes the phenotypic group of blood stasis syndrome.

4.2 Grading of angina pectoris[5]

According to the "Canadian Cardiovascular Society（CCS）grading of angina pectoris", the severity of angina can be graded as follows:

Class I: Ordinary physical activity does not cause angina, such as walking and climbing stairs. Angina with strenuous or rapid or prolonged exertion at work or recreation.

Class II: Slight limitation of ordinary activity. Walking or climbing stairs rapidly, walking

uphill, walking or stair climbing after meals, or in cold, in wind or under emotional stress, or only during the few hours after awakening. Walking more than two blocks on the level and climbing more than one flight of ordinary stairs at a normal pace and in normal conditions.

Class III: Marked limitation of ordinary physical activity. Walking one or two blocks on the level and climbing one flight of stairs in normal conditions and at normal pace.

Class IV: Inability to carry out any physical activity without discomfort; angina may be present at rest.

4.3 Quantification score of angina pectoris[3]

4.3.1 Scoring system

(1) Attack frequency

0 point: None;

2 points: 2-6 episodes per week;

4 points: 1-3 episodes per day;

6 points: At least 4 episodes per day.

(2) Duration

0 point: None;

2 points: Lasting ≤5 minutes per episode;

4 points: Lasting > 5 minutes but < 10 minutes per episode;

6 points: Lasting ≥10 minutes per episode.

(3) Pain intensity

0 point: None;

2 points: Ordinary physical activity does not cause angina, such as walking and climbing stairs. Angina with strenuous or rapid or prolonged exertion at work or recreation;

4 points: Slight limitation of ordinary activity. Walking or climbing stairs rapidly, walking uphill, walking or stair climbing after meals, or in cold, in wind or under emotional stress, or only during the few hours after awakening;

6 points: Marked limitation of ordinary physical activity. Walking one or two blocks on the level and climbing one flight of stairs in normal conditions and at normal pace.

(4) Use of nitroglycerin

a. *frequency of use of sublingual nitroglycerin*

0 point: None;

1 point: Occasionally needed;

2 points: Needed when attack occurs;

3 points: Frequently needed;

b. *time to remission after sublingual nitroglycerin*

0 point: None;

1 point: ≤ 3 minutes;

2 points: >3 minutes but ≤5 minutes;

3 points: >5 minutes;

c. *dose of sublingual nitroglycerin*

0 point: None;

2 points: 1-4 tablets per week;

4 points: 5-9 tablets per week;

6 points: at least 10 tablets per week.

4.3.2 Severity classification

Mild: angina score ≤9 points;

Moderate: angina score 10-18 points;

Severe: angina score ≥19 points.

4.4 Quantification score of TCM syndrome (phenotypic group)[3]

4.4.1 Scoring system

(1) Chest pain: stabbing, gripping, and fixed pain, which radiates to shoulder, back or the medial side of the arm

0 points: none;

2 points: relieved with rest, daily activities not being affected;

4 points: relieved with medication, daily activities not being affected after remission;

6 points: frequent attacks, daily activities being affected (e.g. putting on clothes, eating, walking, or even making a bowel movement may induce symptoms).

(2) Choking sensation in the chest

0 point: none;

1 point: mild chest distress occurs occasionally, which may disappear without any

intervention;

2 points: frequent chest distress, sighing respiration at times, but daily activities not being affected;

3 points: persistent chest suffocation and sighing respiration, daily activities being affected.

(3) Palpitation

0 point: none;

1 point: mild palpitation occurs occasionally, which may disappear without any intervention;

2 points: obvious palpitation occurs sometimes, which may last for a relatively long period, but daily activities can be continued;

3 points: persistent palpitation, which may present even at rest, daily activities being affected.

(4) Dark purple lips

0 point: none;

1 point: light dark lips;

2 points: dark lips with stasis spots;

3 points: dark purple lips with ecchymosis.

(5) Dark purple tongue

0 point: normal or other conditions;

1 point: light dark purple tongue.

2 points: dark purple tongue with stasis spots.

(6) Fine-choppy pulse

0 point: normal or other conditions;

1 point: fine-choppy pulse.

4.4.2 Severity classification

Mild: TCM syndrome score ≤6 points;

Moderate: TCM syndrome score 6 -10 points;

Severe: TCM syndrome score ≥11 points.

4.5 Eligibility criteria

4.5.1 Inclusion criteria

- Patients who fulfill the diagnostic criteria of stable angina pectoris;
- Patients who fulfill the diagnostic criteria of CCS grading class Ⅰ, Ⅱ or Ⅲ;
- Patients who fulfill the diagnostic criteria of blood stasis syndrome;
- Patients who have angina attacks ≥2 episodes per week;
- Ischemic changes on ECG: ST segment depression ≥ 0.05mv and/or deep T wave inversions > 0.2mv, flat T wave less than 1/10R, or positive exercise stress test.
- Aged 40-70 years.
- Those who are willing to provide a signed informed consent form.

4.5.2 Exclusion criteria

- Those who do not fulfill the inclusion criteria;
- Those who have documented acute myocardial infarction or other heart diseases, severe neurosis, menopausal syndrome, hyperthyroidism, cervical spondylosis, gallbladder-heart disease, or gastroesophageal reflux;
- Those who are complicated with poorly-controlled hypertension (systolic BP ≥160mmHg, and/or diastolic BP ≥100mmHg), severe cardiac or pulmonary insufficiency, severe arrhythmia (e.g. rapid atrial fibrillation, atrial flutter, paroxysmal ventricular tachycardia), serious primary diseases of liver, kidney or hematopoietic system, or psychosis;
- Those who have obtained a complete revascularization after coronary artery bypass grafting or coronary intervention;
- Pregnant or lactating women;
- Those who are prone to allergies or with known allergy to many drugs;
- Those who participated in other clinical trials within 1 month prior to randomization.

4.5.3 Withdrawal/dropout criteria

- Those who did not fulfill the eligibility criteria but were included by mistake;
- Those who fulfilled the eligibility criteria but did not take any study drug after randomization;
- Those with poor compliance that might interfere with the efficacy and safety evaluation;

- Those who experienced serious AEs, complications or special physiological changes, making it inappropriate to continue with the trial;
- Individual cases that were unblinded prematurely;
- Those who discontinued the trial voluntarily;
- Those who used any disallowed concomitant medication, especially those that had an obvious effect on the study drug and might interfere with the efficacy and safety evaluation;
- Those who discontinued or lost to follow-up for any other reasons or died during the trial;
- Those with incomplete data that might interfere with the efficacy and safety evaluation.

For all withdrawal/dropout cases, the potential reasons must be recorded. Patients who experienced any AE during the trial must be included in the statistical analysis of adverse drug reactions. Those who have taken the study drug for at least 1 week should be included in the statistical analysis of drug efficacy.

4.5.4 Criteria for study termination

Circumstances that may warrant termination include, but are not limited to:

- Identification of unexpected, significant, or unacceptable risk to subjects;
- Poor efficacy or no efficacy of the study drug;
- Major mistakes in the trial protocol;
- Insufficient adherence to protocol requirements;
- Upon sponsor’s request (e.g. economic or administrative reasons).

4.5.5 Criteria for subject discontinuation

- Any allergic reaction or AE occurs such that continued participation in the study would not be in the best interest of the subject at the discretion of the treating physician;
- Worsening of clinical conditions occurs such that continued participation in the study would not be in the best interest of the subject at the discretion of the treating physician; considered as ineffective cases;
- Subjects are free to withdraw from participation in the study at any time upon

request.

In any case, every effort must be made to determine why patients discontinued the study treatment prematurely, which may include but are not limited to poor confidence in efficacy, occurrence of AEs, SAEs, or other serious complications, worsening of symptoms such that emergent intervention is required. As for those who withdrew from participation in the study or lost to follow-up, it is essential to investigate the potential reasons via telephone or mail. Investigators should record in detail when and how the last dose was administered, the efficacy and safety parameters at and after discontinuation, the relationship between discontinuation or withdrawal and the study drug, and the potential impact of discontinuation cases on the final conclusion of the study. Moreover, case report form (CRF) of withdrawal/dropout cases should also be completed and their original data should also be documented and stored in a secure manner.

5 TEARTMENT REGIMEN

5.1 Origins of study drugs

Trial drug: XXK capsule, manufactured by Chengdu Di'ao Pharmaceutical Group Co. Ltd., Lot No.: 200807081; Approval No.: Z10910051

Control drug: CDS tablet, manufactured by Chengdu Di'ao Pharmaceutical Group Co. Ltd., Lot No.: 081002

Placebo of CDS tablet: manufactured by Chengdu Di'ao Pharmaceutical Group Co. Ltd., Lot No.: 081001

Placebo of XXK capsule: manufactured by Chengdu Di'ao Pharmaceutical Group Co. Ltd., Lot No.: 200807081

5.2 Packaging of study drugs

All drugs used in the study are packaged and supplied by the sponsor in a randomized and blinded manner.

(1) Strength

XXK capsule: 100mg steroidal saponins/capsule,10 capsules/blister × 2blisters/box

CDS tablet: 0.25g×60 piles/bottle

(2) Labeling

Approval number: Z10910051

Study site No.: **Drug No.:**

For use of post-marketing study of Xinxuekang capsule

Functions and indications: promoting blood circulation and removing blood stasis, removing obstruction from the meridians and collaterals, and relieving pain. Coronary heart disease with angina pectoris (blood stasis syndrome) is indicated.

Dosage and administration: for oral use, 1 or 2 capsules a time, 3 times a day.

Duration of treatment: 20 weeks.

Strength: 10 capsules/blister × 2blisters/box

Storage: sealed and stored at the room temperature

Lot No.: 200807081 **Storage life:** 2 years

Supplied by: Chengdu Di'ao Pharmaceutical Group Co. Ltd.

An emergency envelop is inserted into each package, indicating the actual drug name and treatment allocation; and information on how to store this envelop and when to open it is provided on the envelop surface. The distribution process of the study drugs should be well-documented.

5.3 Accountability procedures for study drugs

Upon receipt of the study drugs from the sponsor, investigator or designated individual at each study site should sign a "Receipt form of study drugs" to ensure that the information on the packing slip matches exactly with what has been sent to the site, including the amount, lot numbers and quantity. When an eligible subject is included, investigator or designated individual will dispense the proper drugs in strict accordance with the randomization list. Each time a study drug is dispensed, there should be a "Drug dispensing form" as to the amount dispensed, to whom it is dispensed, and the date and signature of the person dispensing the drug.

Subjects should be advised to return all unused drugs at each study visit. Study personnel should record the amount (number of capsules) and date of return. Study drug will be properly accounted for and tracked with adequate documentation so as to assess subject's compliance.

The study drugs should be stored in a dry and ventilated place at room temperature.

5.4 Study treatment

5.4.1 Drug administration

(1) trial group: XXK capsule + placebo of CDS tablet, oral, 2 capsules + 2 tablets a time, 3 times a day;

(2) control group: CDS tablet + placebo of XXK capsule, oral, 2 tablets + 2 capsules a time, 3 times a day.

5.4.2 Duration

20 weeks treatment.

5.4.3 Concomitant medications

- Sublingual nitroglycerin is permitted if necessary, also with detailed documentation;
- Other TCM or WM medications targeting the treatment of angina or CHD will be disallowed during the trial;
- If other medication or therapy is required to treat the concomitant diseases, then the name of the drug or therapy, actual dosage, dosing frequency and start/stop time should be well-documented.

6 OBSERVATION INFORMATION

6.1 Safety parameters

- Vital signs;
- Blood, urine and stool routine tests;
- Liver function (ALT), kidney function (BUN and Cr);
- Any AE that occurs throughout the treatment period.

The first 3 items will be observed or evaluated before and after treatment.

6.2 Efficacy parameters

The primary efficacy endpoint of this study is:

- Proportions of patients who were angina-free.

Angina-free was defined as the responses of patients' anginal symptoms were”none”on the Quantification Score of Angina Pectoris(see page19 for details) including three dimensions (attack frequency, severity and duration of angina) over the 20-weeks treatment phase. The Quantification Score of Angina Pectoris comprising questions investigating the patients' anginal symptoms was administered by the physician in charge of the study at 2, 4, 6, 8 and 20 weeks in each center.

- Proportion of effective electrocardiogram (ECG) improvement.

The effective ECG improvement was defined as a standard 12-lead ECG returns to normal(or within the normal range) according to the International Society and Federation of Cardiology/World Health Organization after 20-weeks long term treatment. The ECG was performed at 0,8, and 20 weeks during treatment.

Secondary efficacy endpoints are:

- Change from baseline in weekly average frequency of self-reported angina episode over the 20-weeks treatment phase.
- Weekly average nitroglycerin consumption rate at week 8 and week 20.

Throughout the study, patients recorded the occurrence of anginal attacks and the number of nitroglycerin use on diary cards and the study staff at each clinical site reviewed the angina and nitroglycerin use cards with the patient at each study visit(2, 4, 6, 8 and 20 weeks) to ensure accuracy.

- Change from baseline in 5 dimensions of the Seattle Angina Questionnaire (SAQ) and the proportions of patients who were clinical significance at week 8 and week 20.

The SAQ is a disease-specific functional status measure that was developed to quantify the physical and emotional effects of CAD[6].Each SAQ dimension (anginal frequency, physical limitation, anginal stability, disease perception, and treatment satisfaction) was scored on a scale of 0 to 100 and the clinical significance was defined as a difference of 8 points or more on the physical-limitation scale, 25 or more on the angina-stability scale, 20 or more on the angina-frequency scale, 12 or more on the treatment-satisfaction scale, and 16 or more on the quality-of-life scale[7].Patients will complete the SAQ at 0,8, and 20 weeks.

- Change from baseline in the blood stasis syndrome and the proportion of patients who had significant syndrome improvement.

Blood stasis syndrome which a common syndrome of chronic stable angina in TCM including the following symptoms: angina, choking sensation in the chest, palpitation, dark purple lips, ecchymosis on the tongue, and fine-choppy pulse.The syndrome score was assessed by using the Quantification score of TCM syndrome(with higher scores indicating higher severity, see page20 for details)and administered by the physician in charge of the study at 2, 4, 6, 8 and 20 weeks in each center.

The significant syndrome improvements defined as clinical signs and symptoms have been greatly improved, and decrease in syndrome score ⩾70%[3].

- Change from baseline in ECG exercise test (the first 1/3 of patients included at each site)performed at 0 and 8 weeks.

Investigators should be aware of the contraindications to exercise test, and patients should be closely monitored during the test to ensure his/her safety, including ECG, BP, and angina.

- Change from baseline in serum lipid: total cholesterol, triglyceride, HDL cholesterol, and LDL cholesterol, measured at 0 and 8 weeks during treatment.
- Related signs: heart rate, rhythm, murmur, and BP, recorded before and after Treatment.
- Accompanying symptoms after treatment: fatigue, shortness of breath, aversion to cold, cold extremities and etc. (recorded as “yes” or “no”).

7 EFFICACY EVALUATION

7.1 Criteria for overall efficacy evaluation

- Markedly effective: main symptoms like angina disappear or almost disappear, and ECG returns to normal (or within the normal range);
- Effective: main symptoms like angina and ECG features have been improved;
- Ineffective: main symptoms like angina and ECG features remain unchanged;
- Worsening: main symptoms like angina and ECG features become worse.

If the changes in main symptoms are inconsistent with those in ECG features, that of lower efficacy shall prevail.

7.2 Evaluation of angina changes

The evaluation of angina changes should be based on the quantification score of angina pectoris:

- Markedly effective: angina symptoms disappear or almost disappear, and decrease in angina score ≥70%;
- Effective: attack frequency, severity and duration of angina have been greatly improved, and decrease in angina score ≥30% but <70%;
- Ineffective: angina symptoms remain unchanged, and decrease in angina score

<30%;

- Worsening: angina symptoms become worse, and decrease in angina score <0.

7.3 Evaluation of syndrome changes

The evaluation of syndrome changes should be based on the quantification score of the TCM syndrome:

- Markedly effective: clinical signs and symptoms have been greatly improved, and decrease in syndrome score ≥70%;
- Effective: clinical signs and symptoms have been somewhat improved, and decrease in syndrome score ≥30% but <70%。
- Ineffective: clinical signs and symptoms remain unchanged, and decrease in syndrome score <30%;
- Worsening: clinical signs and symptoms become worse, and decrease in syndrome score <0.

The dynamic changes in the phenotypic group should also be assessed.

7.4 Evaluation of ECG changes

- Markedly effective: ECG returns to normal (or within the normal range);
- Effective: reversion of S-T segment depression ≥ 0.05mV but still remains out of the normal range, improvement in deep T wave inversions (≥ 25%) or transformation of flat T wave into upright T wave, and improvement in atrioventricular or intraventricular block;
- Ineffective: ECG features remain unchanged;
- Worsening: aggravation of S-T segment depression ≥ 0.05mV, deeper T wave inversions (≥ 25%) or transformation of upright T wave into flat T wave or flat T wave into inverted T wave, identification of ectopic rhythms, atrioventricular or intraventricular block.

7.5 Evaluation of changes in symptoms or signs

- Markedly effective: clinical signs and symptoms disappear or 2 points lower relative to baseline (e.g. 3 points to 1 point);
- Effective: clinical signs and symptoms have been improved or 1 point lower relative to baseline (e.g. 3 points to 2 points, or 2 points to 1 point);

- Ineffective: clinical signs and symptoms remain unchanged;
- Worsening: clinical signs and symptoms become worse.

7.6 Use of nitroglycerin

- Withdrawal: complete withdrawal after treatment;
- Dose reduction: the number of doses decreased at least 50% after treatment;
- Unchanged: the number of doses decreased less than 50% after treatment.

8 ADVERSE EVENTS

8.1 Background information about drug-related safety issues

Based on the data from preclinical pharmacological and toxicological studies provided by the sponsor, evidence of XXK-related adverse reactions is not identified.

8.2 Monitoring and recording of adverse events

8.2.1 Definition

Adverse event (AE): An AE is any untoward medical occurrence in a patient or clinical investigation subject administered a pharmaceutical product and that does not necessarily have a causal relationship with this treatment. An AE can therefore be any unfavorable and unintended sign (including an abnormal laboratory finding), symptom, or disease temporally associated with the use of a medicinal (investigational) product, whether or not related to the medicinal (investigational) product. Pre-existing conditions which worsen during a study are also to be reported as AEs.

Serious Adverse Event (SAE): A SAE is any experience that suggests a significant hazard, contraindication, side effect or precaution. It is any AE that at any dose fulfils at least one of the following criteria:

– is fatal;

– is life-threatening;

– required in-patient hospitalization or prolongation of existing hospitalization;

– results in persistent or significant disability/incapacity;

– is a congenital anomaly/birth defect;

– is medically significant or requires intervention to prevent one or other of the outcomes listed above.

Adverse drug reaction (ADR): An ADR is any unintended harm or injury associated

with the use of given medications at a normal dosage during normal use. In clinical trials of any new drug or any new usage of a certain drug, any unintended harm or injury that has a causal relationship to the use of the drug should also be considered as an ADR.

8.2.2 Recording

For all AEs encountered during the clinical study, the following must be assessed and recorded on the adverse events form of the case report form (CRF): intensity, relationship to study drug, action taken regarding study drug, and outcome to date. All AEs, especially those for which the relationship to test drug is not "unrelated", should be followed up until they have returned to baseline status or stabilized. The follow-up procedures could be performed in clinic visits or during hospitalization, or via home visit, telephone or mail, depending on the severity of AEs.

8.3 Severity of An AE

Mild: events require minimal or no treatment and do not interfere with the subject's daily activities.

Moderate: events result in a low level of inconvenience or concern with the therapeutic measures. Moderate events may cause some interference with functioning.

Severe: events interrupt a subject's usual daily activity and may require systemic drug therapy or other treatment. Severe events are usually incapacitating.

8.4 Relationship to study drugs

Attribution of an AE can be categorized as not related, unlikely related, possibly related, probably related, or definitely related to the study drugs.

Not related: The AE is clearly not related to the investigational agent/procedure. - i.e. Another cause of the event is most plausible; and/or a clinically plausible temporal sequence is inconsistent with the onset of the event and the study medication administration; and/or a causal relationship is considered biologically implausible.

Unlikely related: The AE is doubtfully related to the investigational agent/procedure.

Possibly related: The AE follows a reasonable temporal sequence from administration of the study drug/procedure, follows a known or expected response pattern to the suspected drug, but that could readily have been produced by a number of other factors.

Probably related: The AE is likely related to the investigational agent/procedure.

Definitely Related: The AE is clearly related to the investigational agent/procedure – i.e. An event that follows a reasonable temporal sequence from administration of the study drug, follows a known or expected response pattern to the suspected drug, that is confirmed by improvement on stopping and reappearance of the event on repeated exposure and that could not be reasonably explained by the known characteristics of the patient’s clinical state.

8.5 Handling of SAEs

All SAEs should be managed properly and timely, and recorded on the appropriate SAE CRF. Any AE considered serious by the investigator or which meets the aforementioned criteria must be submitted on a signed and dated SAE form to the sponsor, ethics review committee and relevant authorities within 24 hours of site awareness. And the sponsor should also inform all study sites of such SAEs in a timely manner to ensure subjects’ safety in accordance with applicable laws and regulations.

Fatal or life-threatening events considered to be caused by the study drugs must be reported immediately to the sponsor and relevant authorities via telephone, fax, EMS or E-mail.

8.6 Opening and handling of emergency envelops

The emergency envelope provided along with each test drug in its package should only be opened in case of a serious complication or a serious adverse event, which is called emergency unblinding. Once unblinded, the patient should be withdrawn from the study and regarded as a dropout case. Information about when, where and why the emergency unblinding occurs as well as each personnel’s signature should also be documented.

8.7 Follow-up of unresolved AEs

All AEs should be followed up until they have been resolved, stabilized or returned to baseline status.

9 SAFETY EVALUATION

Level 1: safe, no ADRs;

Level 2: relatively safe, treatment continues without any intervention;

Level 3: safety issues observed, but treatment can be continued after proper intervention;

Level 4: study termination due to ADRs.

10 DATA MANAGEMENT

10.1 Collection and fulfillment of CRF

The investigators fill in the 3-copy CRF of every patient enrolled in a complete, accurate, and timely manner. After the monitors review the completed CRF, the first copy of the CRF will be sent to the statisticians for data collection and management. Once the first copy of the CRF is submitted, the CRF will not be done any modification.

10.2 Data collection and management

The statisticians are responsible for data collection and management. Data management will be performed via EpiData 3.0. To assure the accuracy of the data, two statisticians should record the data from the CRF independently. Once there are any queries about the CRF, the statisticians should fill in a data queries form (DQF), and send it to the monitors. Then the monitors require the investigators to resolve the queries as soon as possible. The statisticians will do the data modification according to the correspondence from the investigators. The statisticians could send another DQF if necessary.

10.3 Data Lock

After confirming the accuracy of the database established from the CRF, the investigators, sponsor, statisticians will agree to lock the data. The locked database should not be done any modification. Any problems found after the data lock will be resolved in the procedure of statistical analysis.

11 STATISTICAL CONSIDERATIONS AND ANALYTICAL PLAN

11.1 Selection of data sets

11.1.1 Efficacy analysis sets

Full analysis set (FAS): includes all randomized patients and those without any evaluable follow-up data after randomization are excluded. For patients who did not complete the study according to the protocol, a last observation carried forward (LOCF) procedure was performed.

Per protocol set (PPS): includes all subjects in the FAS population who meet the study treatment compliance criteria and had no major protocol violations.

For primary outcome measures, statistical analyses will be performed separately in both FAS and PPS populations.

11.1.2 Safety analysis set

The safety analysis set (SS) is defined as a subset of subjects who took at least 1 dose of study drug after randomization.

11.2 Dynamic statistical plan

One interim analyses will be conducted under blinding conditions after 1/2 of the total number of cases have completed the trial.

Statisticians and principal investigators are responsible for drafting a written plan for statistical analysis according to the study protocol. It includes the number of included, excluded or withdrawal patients, the demographic and baseline characteristics of included patients, treatment compliance, efficacy analysis and safety analysis.

Qualitative data will be described with absolute and relative (proportion or percentage) values; and quantitative data will be summarized with mean, standard deviation, maximum, minimum, and median.

Paired t-test will be used for comparison before and after treatment within a treatment group. In analysis of variance (ANOVA) for multiple comparisons within a group, if the variances are assumed to be equal, then Student-Newman-Keuls (SNK) significance test will be used for pairwise comparison; if the variances are not assumed to be equal, then non-parametric test will be applied. Two-samples t-test will be used for comparison between treatment groups. Fourfold table will be displayed for comparison of categorical parameters: Chi-squared test for nominal categorical data and rank sum test for ordinal categorical data. Also, the analysis of covariance (ANCOVA) can be used to control for potential confounding variables.

All *P* values are two-side. Statistical significant level is set to be 0.05.

For between-group comparisons, chi-square test, Fisher's exact test, Wilcoxon rank test or Cochran-Mantel-Haenszel (CMH) χ^2 test may be used for qualitative data; while t-test, ANOVA, Wilcoxon rank test or ANCOVA may be applied for quantitative data.

Baseline analysis: Demographic and baseline characteristics will be reported for each subject. Comparisons between the treatment groups will be conducted using ANOVA or

Chi-square test to assess the degree to which comparability of randomization can be achieved.

Dropout analysis: The overall dropout rate and the dropout rate due to AEs will be compared between the treatment groups by using Chi-square test.

Efficacy analysis: The effect of different study sites on efficacy parameters will be considered and analyzed.

Dynamic change of TCM syndrome (phenotypic group): The method of Multidimensional Scaling Analysis (MDS) will be used to investigate the changing tendency of the phenotypic group at different time points in different treatment groups. Different dimensionality values of any patients at different time points make up a vector which is called control vector; and the maximum values of the quantification score of six phenotypes make up another vector, which is called standard vector. The included angle cosine method commonly used in Similarity Degree Analysis (SDA) is adopted to analyze the similarity degree of different patients at different time points. Nonlinear mixed effect model (NONMEM) is built to analyze the nonlinear relationship between the TCM syndrome (phenotypic group) and observation time points, also to identify the cutoff point in the dynamic change of the TCM syndrome (phenotypic group).

Safety analysis: Safety will be evaluated by tabulations of adverse events and will be presented with descriptive statistics at baseline and follow-up visits for each treatment group. Adverse event incidence rates will be summarized by system organ class, preferred term, and severity of the adverse event All information pertaining to adverse events noted during the study will be listed by subject, preferred term, system organ class, date of onset, date of resolution, severity, and relationship to study drug.

12 QUALITY CONTROL AND QUALITY ASSURANCE

12.1 Laboratory quality control

The laboratories at each study site should have standard operating procedures (SOPs) and quality control procedures.

12.2 Investigator training

All investigators at each study site should attend a training session before participation to have a deep understanding of all the requirements in the conduct of the clinical trial.

12.3 Compliance enhancement

The informed consent procedure is essential to ensure patients' compliance to treatment. The study drug, laboratory examinations, transportation and healthcare consultation will be provided by the sponsor free of charge.

Compliance rate is calculated by taking the amount of drug ingested divided by the amount the patient should have ingested and multiply by 100%. A compliance rate at 80%-120% defines good compliance; while that < 80% or > 120% defines poor compliance.

Patients should be asked to report all the concomitant medications while on study. Patients who have poor efficacy or poor compliance should be closely followed up.

12.4 Study monitoring

Clinical Study Coordinators (CSCs) will be designated to conduct on-site monitoring during the trial. The main responsibilities of CSC include:

- Assure the protection of the rights, safety and well being of study subjects.
- Analyze and evaluate clinical data, to ensure investigator and site compliance with the study drug protocol, overall clinical objectives, and applicable regulations.
- Identify, help in the study site selection process, initiate, and eventually close out clinical study sites.
- Monitor the progress of clinical study sites and assure the protocol is followed and data is reported accurately.
- Make certain that the scientific integrity of the data collected is protected and verified.
- Assure that adverse events are correctly documented and reported.
- Review all case report forms and compare them to source documents.

12.5 Multi-center study coordination committee

A multi-center study coordination committee is set up to further ensure the quality of the clinical trial, which includes a Data Monitoring Committee (DMC) and a Implementation & Decision-making Committee. The multi-center study coordination committee is made up of the principal investigators, leaders of each study site and representatives of the sponsor.

13 ETHICAL ASPECTS

The investigator will ensure that this study is conducted in full conformance with the principles of the "Declaration of Helsinki" and applicable laws and regulations on the conduct of clinical trials.

This protocol and any accompanying material provided to the subject will be submitted by the investigator to the Ethics Committee of the Institute of Basic Clinical Research, China Academy of Chinese Medical Sciences. Approval from the board must be obtained before starting the study.

Informed consent is a process that is initiated prior to the individual's agreeing to participate in the study and continues throughout the individual's study participation. Extensive discussion of risks and possible benefits of this treatment will be provided to the subjects and their families. Consent forms describing in detail the study interventions/products, study procedures, and risks are given to the subject and written documentation of informed consent is required prior to starting intervention/administering study product. It must also be explained to the patients that they are completely free to refuse to enter the study or to withdraw from it at any time for any reason. A copy of the informed consent document will be given to the subjects for their records.

14 STUDY DOCUMENTATION, CRFS AND RECORD KEEPING

14.1 Investigator's files/retention of documents

The investigator must maintain adequate and accurate records to enable the conduct of the study to be fully documented and the study data to be subsequently verified. These documents should be classified into two different separate categories: investigator's study file, and patient clinical source documents.

The investigator's study file will contain the protocol/amendments, a paper representation of the CRF, Ethics Committee and governmental approval with correspondence, sample informed consent, drug records, staff curriculum vitae and authorization forms and other appropriate documents/correspondence etc. Patient clinical source documents would include patient hospital/clinic records, physician's and nurse's notes, appointment book, original laboratory reports, ECG, and special assessment reports, signed informed consent forms, consultant letters, and subject screening and

enrollment logs.

In all sites the investigator must keep these two categories of documents (including the archival compact disk) on file for at least 10 years after completion or discontinuation of the study. After that period of time the documents may be destroyed once the site has provided written notification 60 days prior to destruction of the documents, subject to local regulations. No records should be disposed of without the written approval of the sponsor.

14.2 Source documents and background data

CSCs will perform ongoing source data verification to confirm that critical protocol data (i.e., source data) entered on the CRFs by authorized site personnel are accurate, complete, and verifiable from source documents. In no case is the CRF to be considered as source data for this trial.

The investigator shall supply the sponsor on request with any required background data from the study documentation or clinic records. This is particularly important when errors in data transcription are suspected. In case of special problems and/or governmental queries or requests for audit inspections, it is also necessary to have access to the complete study records, provided that patient confidentiality is protected.

Source documents that are required to verify the validity and completeness of data transcribed on the CRFs must never be obliterated or destroyed.

14.3 Audits and inspections

The investigator should understand that source documents for this trial should be made available to appropriately qualified personnel from the sponsor's quality assurance group or its designers or to health authority inspectors after appropriate notification. The verification of the CRF data must be by direct inspection of source documents.

14.4 Case Report Forms

Data for this study will be managed on computer via Epidata 3.0 from Case Report Forms. The data is entered on to the computer using the off-line mode. An audit trail will maintain a record of initial entries and changes made; reasons for change; time and date of entry; and user name of person authorizing entry or change. The investigator will connect on a regular basis, using an analog phone line, and the data will be transferred directly to the Sponsor's database.

For each patient randomized, a CRF must be completed and signed by the principal investigator or authorized delegate from the study staff. This also applies to records for those patients who fail to complete the study. If a patient withdraws from the study, the reason must be noted on the CRF. If a patient is withdrawn from the study because of a treatment-limiting AE, thorough efforts should be made to clearly document the outcome.

The investigator should ensure the accuracy, completeness, and timeliness of the data reported to the sponsor in the CRFs and in all required reports.

15 CLINICAL TRIAL FLOWCHART

Clinical Trial Flowchart

Stages	Recruitment	Treatment Phase				
Visits	1st	2nd	3rd	4th	5th	6th
Time	Before treatment (Week 0)	Week 2	Week 4	Week 6	Week 8	Week 20
Informed consent	√					
Medical history	√					
Eligibility criteria	√					
Randomization	√					
Drug distribution	√	√	√	√	√	√
Patient general characteristics	√					
Specialty examination	√	√	√	√	√	√
TCM syndrome scoring	√	√	√	√	√	√
Blood routine	√				√	√
Urine routine	√				√	√
Stool routine	√				√	√
Liver function	√				√	√
Kidney function	√				√	√
Blood lipid	√				√	

ECG	√				√	√
Recording of concomitant medication	√	√	√	√	√	√
Recording of AEs		√	√	√	√	√
Quantification Score of Angina Pectoris	√	√	√	√	√	√
Seattle Angina Questionnaire(SAQ)	√				√	√
Review of angina frequency, nitroglycerin use	√	√	√	√	√	√
ECG exercise test	√				√	

16 PUBLICATION POLICY

The results of this study may be published or presented at scientific meetings. If this is foreseen, the investigator agrees to submit all manuscripts or abstracts to the Sponsor prior to submission. This allows the sponsor to protect proprietary information and to provide comments based on information from other studies that may not yet be available to the investigator.

In accord with standard editorial and ethical practice, the sponsor will generally support publication of multicenter trials only in their entirety and not as individual center data. In this case, a coordinating investigator will be designated by mutual agreement.

Any formal publication of the study in which input of the sponsor personnel exceeded that of conventional monitoring will be considered as a joint publication by the investigator and the appropriate the sponsor personnel. Authorship will be determined by mutual agreement.

17 REFERENCES

1. Weintraub WS, Spertus JA, Kolm P, et al. Effect of PCI on Quality of Life in Patients with Stable Coronary Disease. N Engl J Med 2008;359:677-87.

2. Feng ZY. Phase II clinical trial of Di'ao Xinxuekang Capsule in treating angina pectoris.

New Drugs and Clinical Remedies1994; 13(3):152-155

3. Zheng Xiaoyu. Guidance principle of clinical study on new drug of TCM. Beijing: China Medico-Pharmaceutical Science and Technology Publishing House, 2002: 68-73.

4. Nomenclature and criteria for diagnosis of ischemic heart disease. Report of the Joint International Society and Federation of Cardiology/World Health Organization task force on standardization of clinical nomenclature. Circulation1979;59(3):607-9.

5. Campeau L. Grading of angina pectoris. Circulation 1976;54:522–3.

6. Spertus JA, Winder JA, Dewhurst TA, et al. Monitoring the qualityeof life in patients with coronary artery disease. Am J Cardiol1994;74:1240–4.

7. Tierney WM, Babu AN, Wolinsky FD. Clinically important differences in health status for patients with heart disease: an expert consensus panel report. Am Heart J 2004;147:615-22

18 CONDITIONS FOR MODIFYING THE PROTOCOL

Protocol modifications to ongoing studies must be made only after consultation between an appropriate representative of the sponsor and the investigator representatives. Protocol modifications must be prepared by a representative of the sponsor and initially reviewed and approved by the principal investigators and statisticians.

All protocol modifications must be submitted to the Ethics Committee for information and approval in accordance with local requirements, and to regulatory agencies if required. Approval must be awaited before any changes can be implemented, except for changes necessary to eliminate an immediate hazard to trial patients, or when the changes involves only logistical or administrative aspects of the trial, e.g. change in monitors, or change of telephone numbers.
